# Affective Forecasting Accuracy in Everyday Life

**DOI:** 10.1007/s42761-026-00364-x

**Published:** 2026-03-07

**Authors:** Ella K. Moeck, Komal K. Grewal, Ashish Mehta, Katharine H. Greenaway, Peter Koval, Elise K. Kalokerinos

**Affiliations:** 1 School of Psychology, Adelaide University, Adelaide, Australia; 2 Melbourne School of Psychological Sciences, The University of Melbourne, Melbourne, Australia; 3 School of Psychology, Deakin University, Melbourne, Australia; 4 School of Psychology, Stanford University, Stanford, United States of America

**Keywords:** Affective forecasting, Predicted emotions, Forecasting errors, Unpleasant events

## Abstract

People often predict how they might feel in the future, with varying degrees of accuracy. Such *affective forecasts* can centre around periods of time (e.g., tomorrow, next week) and/or specific events (e.g., an upcoming meeting). Affective forecasts for everyday events or periods of time are the building blocks of everyday decision-making. Yet, most affective forecasting research has focused on forecasting accuracy for rare and consequential events, such as election results and romantic break-ups. Therefore, in two intensive longitudinal datasets, we tested everyday forecasting accuracy in general (e.g., “tomorrow”) and for specific unpleasant events. In Study 1—a week-long experience sampling study—participants (*N* = 209) provided (i) one weekly forecast about their feelings over the next week, and (ii) daily forecasts about their feelings the next day. Participants also rated their (i) daily affect each evening, and (ii) their momentary affect nine times each day. In Study 2—a two-week daily diary study—participants (*N* = 69) nominated an upcoming unpleasant event each day and forecasted their affect related to that event. Each evening, participants rated how they felt when this event occurred. We found that participants could predict when a day/event would make them feel better/worse than usual, showing relative accuracy, but sometimes made small errors in forecasting their absolute affect levels, showing absolute inaccuracy. These findings suggest people make smaller forecasting errors in everyday life than for major events, which likely aids everyday decision making, for example by informing the use of future-oriented regulation strategies.

**Supplementary Information:**

The online version contains supplementary material available at 10.1007/s42761-026-00364-x.

How accurately can people predict how they will feel in a day, a week, or in relation to an upcoming event in their daily lives? People often think about the future (Beaty et al., 2019) and base decisions on their predicted feelings, or *affective forecasts* (Carlson et al., 2023; DeWall et al., 2016; Ferrer et al., 2015). While several studies have investigated forecasting accuracy for specific, circumscribed, and often rare events (e.g., election results), research investigating everyday affective forecasting is scarce. Arguably, forecasts for specific everyday events matter more for everyday decision-making than forecasts for rare events (Patel & Urry, 2024). Such everyday affective forecasts (*“Tonight’s party is going to make me feel sad”*) may underpin future-oriented affect-regulation strategies, such as situation selection (deciding not to go to the party) or situation modification (inviting a friend to the party; Floerke et al., 2017). Thus, we used intensive longitudinal methods to investigate accuracy in people’s forecasts of their day-to-day positive and negative affect^1^ in general (Study 1) and for specific daily unpleasant events (Study 2).

Affective forecasting refers to people’s predictions about how they will feel in the future (e.g., Wilson & Gilbert, 2003). Forecasting accuracy is usually examined by comparing how people predict they will feel when a specific event occurs (*forecasted affect*) to how they actually feel when this event occurs (*experienced affect*). Early research suggested affective forecasts are often inaccurate, such that people tend to overestimate the intensity and/or duration of affective responses to future events, known as the *impact bias* (see Wilson & Gilbert, 2003). The tendency to overestimate the affective impact of events has been demonstrated across several contexts, including medical (Bosch et al., 2021), political (e.g., Lench et al., 2019), and educational (Finkenauer et al., 2007). But other studies show that people sometimes underestimate their future affect—including underestimating positive feelings when interacting with strangers (e.g., Epley & Schroeder, 2014) and negative feelings when socially excluded (Nordgreen et al., 2012)—or can make accurate forecasts (e.g., Levine et al., 2012). Thus, while overestimation is the predominant finding, there is also evidence of people underestimating and accurately forecasting their affect.

These findings aside, by focusing on forecasting accuracy for rare events, researchers have overlooked affective forecasts of other kinds. To guide everyday behavior and decision-making, people need to be able to predict how they will feel over the near-term (e.g., tomorrow, next week) or about daily events (e.g., an upcoming work meeting, a get-together with friends). For instance, if you think you will feel anxious tomorrow, then you might schedule a gym class to stave off the anxiety. It is therefore important to understand what affective forecasting accuracy looks like in daily life. A handful of studies have investigated the accuracy of general affective forecasting in daily life, but have mixed findings and limitations. Colombo et al. (2020) asked participants to forecast positive and negative affect “over the next two weeks” and found that participants tended to *underestimate* their average momentary positive and negative affect level. By contrast, Mathersul and Ruscio (2020) and Wenze et al. (2012) both found for weekly forecasts that participants tended to *overestimate* positive and negative affect. One possible explanation for these mixed findings is that these studies cover relatively long time-windows (i.e., 1–2 weeks). But affect is highly dynamic (Kuppens & Verduyn, 2017). While people should expect their affect to vary considerably during a one to two week period, they may be more accurate in forecasting their affect over shorter timescales, such as a single day. To address this limitation and provide a true estimate of forecasting accuracy in everyday life, we need to measure forecasted and experienced affect over both long (e.g., one week) and short (e.g., one day) timescales.

## The Current Research

We aimed to characterise affective forecasting accuracy in everyday life using data from two intensive longitudinal studies. Study 1 was a 7-day experience sampling study, where participants repeatedly forecasted their positive and negative affect for the next day as well as their affect for the next week. Study 2 was a 14-day daily diary study, where participants forecasted their positive and negative affect in response to a specific unpleasant event that they expected to occur each day. We post-registered both studies (Benning et al., 2019), meaning research questions, hypotheses, and analysis plans were registered after data collection but before the first author accessed the data.

We assessed affective forecasting accuracy in absolute and relative terms (Mathieu & Gosling, 2012). *Absolute accuracy* (or level convergence; Neubauer et al., 2020) refers to the difference in magnitude between forecasted and experienced affect, and is predominantly how researchers have conceptualized forecasting accuracy. For example, with affect rated on a 100-point scale, a 20-point absolute overestimation occurs if a person forecasts their positive affect as 80/100, but then rates their experienced positive affect as 60/100. By contrast, *relative accuracy* (or correspondence convergence; Neubauer et al., 2020) refers to the correlation between forecasted and experienced affect ratings: i.e., whether forecasting a higher/lower-than-average affect level corresponds with experiencing a higher/lower-than-average affect level. Continuing with our example, if the same person’s typical level of positive affect were 50/100, their forecasted level of 80/100 would reflect *relative* accuracy even if they experienced only 60/100, because they accurately predicted feeling *more* pleasant than usual. A meta-analysis found that affective forecasts tend to be inaccurate in absolute terms, but accurate in relative terms (Mathieu & Gosling, 2012), highlighting the need to focus on both measures of forecasting accuracy.

## Study 1: Daily and Weekly General Affective Forecasts

Our registered hypothesis was that participants would show relative forecasting accuracy (consistent with Mathieu & Gosling, 2012): forecasted positive/negative affect (for tomorrow and next week) would positively predict experienced positive/negative affect. We did not register hypotheses for absolute accuracy, because these analyses were added after registering. We also registered hypotheses and analyses testing whether affective forecasting is associated with emotional benefits (e.g., emotion-focused coping, daily well-being). We focus solely on accuracy in this paper, but report method details and results for these analyses in the Supplemental Material (Supplementary Tables S1, S2, S3, S4, S5, S6).

## Method

### Transparency and Openness

We collected data for this study alongside data for other research questions (Moeck et al., 2023; Sels et al., 2024). We pre-registered the broader data collection procedure (https://osf.io/rwbgj/) on the Open Science Framework. We separately post-registered our analysis plan after data were collected but before they were analyzed (https://osf.io/86fgv/). See https://osf.io/tgb4x for relevant Study 1 data and https://osf.io/qcksn for analysis code. Below, we focus on details relevant to the current research questions.

### Participants

We determined our sample size based on an a priori power analysis, using Murayama et al.’s (2022) method, using parameters (*b =* 0.031; *t* = 3.501; random slope variance, 𝝉_11_=0.011) from a small Level-1 effect observed in an experience-sampling dataset on daily emotional functioning (Grommisch et al., 2020). We needed 171 participants with a cluster size of 63 (9 daily surveys for 7 days) to obtain 80% power of detecting a similar effect as significant at *p* <.05. We aimed for over 200 participants to allow for attrition.^2^

We recruited 237 participants via online ads and the University of Melbourne undergraduate psychology research participation pool. Eligible participants were over 18, located within the Australian Eastern Standard time-zone for the duration of the study, and had a compatible iOS or Android smartphone. We excluded 28 participants: eight failed two attention checks at baseline, seven were located outside the target time-zone, six formally withdrew, two were concurrently completing another experience-sampling study, two experienced technical difficulties, two were aged <18 years, and one completed no experience-sampling surveys.

Our final sample (*N* = 209) comprised mostly general community participants (*n =* 176; university participant pool: *n* = 33) aged 18 to 71 years (*M* = 32.55, *SD* = 10.99). There were 42 men, 165 women, one non-binary participant, and one who did not report their gender. Most participants identified as White/Caucasian (68.3%) or Southeast, South, or East Asian (24.5%).

### Procedure

The study ran for 8 consecutive days. Participants completed an online baseline survey via Qualtrics (Day 0), then experience sampling surveys on their smartphones via the SEMA3 app (O’Brien et al., 2024; Days 1–7). Participants received up to 3% course credit or $65AUD (in vouchers); the amount depended on survey completion rates (reimbursement schedule available: https://osf.io/rwbgj/).

### Baseline Survey

After providing consent, participants completed demographics, several questionnaires, forecasted their affect for the upcoming week, and watched videos outlining the experience-sampling procedure. Embedded throughout baseline were three attention checks: two instructional and one open-ended. Participants who failed at least two attention checks were not invited to the experience-sampling portion of the study.

### Experience Sampling Surveys

Each day, participants completed 9 momentary and one end-of-day survey. End-of-day surveys began on Day 0 and momentary surveys on Day 1. To address careless responding, we replaced any items responded to in < 650ms with missing data (0.89% of momentary, 0.77% of end-of-day items; (Geeraerts & Kuppens, 2020). We replaced entire surveys with missing data (0.01% momentary, 0.007% end-of-day surveys) when participants responded in < 650ms on over half the items.

#### Momentary Surveys

Momentary surveys comprised 25 items; 8 relevant to this study. We used a stratified random-interval scheme and divided the day (9:00–20:25) into nine 45-min intervals separated by 35-min. We sent a survey randomly in each interval (inter-survey interval: *M =* 80.19, *SD* = 19.11 min), with 20-min expiry. Mean compliance—percentage surveys completed of surveys received—was 71.34% (*median =* 77.8, *SD* = 21.92%). There were 9,251 completed momentary surveys.

#### End-Of-Day Surveys

End-of-day surveys comprised 21–25 items; 16 relevant to this study. Items included in the Day 0 and Day 7 surveys varied slightly from the Day 1–6 surveys, as outlined below.^3^ We used a fixed sampling scheme, sending surveys at 21:00 with 23:59 expiry. End-of-day compliance was 89.11% (*median =* 100.00, *SD* = 18.69%). There were 1,468 completed end-of-day surveys.

## Measures

### Positive and Negative Affect

We included four positive (low-arousal: *peaceful*,* relaxed*; high-arousal: *excited*,* enthusiastic*) and four negative (low-arousal: *sad*,* dull*; high-arousal: *anxious*, *irritated*) affect items (Yik et al., 2011). Participants rated each item in random order on a slider scale from 0 (*not at all*) to 100 (*very much*). The question stem varied depending on the timeframe participants rated their affect around (Table 1 includes question wording and scale reliabilities).

**Table 1:** Question wording and reliability estimates for forecasted and experienced affect measures by timeframe and valence (Positive Affect [PA], Negative Affect [NA]) for Study 1 and 2

		Between person reliability		Within person reliability	
	Question stem	PA	NA	PA	NA
*Forecasted affect*					
Weekly forecasted affect (Study 1)	“Overall, how *[affect item]* do you think you will feel **in the next week**?”	*r*=.51	*r*=.50	-	-
Daily forecasted affect (Study 1)	“Overall, how *[affect item]* do you think you will feel **tomorrow**?”	0.90	0.86	0.70	0.62
Event-related forecasted affect (Study 2)	“During **this situation**, I expect I will feel [*affect item*]”	0.73	0.75	0.85	0.65
*Experienced affect*					
Weekly retrospective affect (Study 1)	“Overall, how *[affect item]* did you feel **in the past 7 days**?”	*r*=.78	*r*=.79	-	-
Daily retrospective affect (Study 1)	“Overall, how *[affect item]* did you feel **today**?”	0.86	0.85	0.71	0.65
Momentary affect (Study 1)	“**Right now** how *[affect item]* do you feel?”	0.88	0.86	0.70	0.62
Event-related retrospective affect (Study 2)	“Looking back at my day, **this situation** made me feel [*affect item*]”	0.92	0.87	0.77	0.68

*MultilevelTools* package (Wiley, (2020) used to calculate omega for ESM scales. Correlations (not omega) used to calculate between-person reliability for forecasted weekly affect, due to these scales comprising two items only

For weekly forecasts, participants forecasted affect for the upcoming week in the baseline survey (*weekly forecasted affect*) and rated their experienced affect for the past week in the Day 7 end-of-day survey (*weekly retrospective affect*). To reduce baseline survey length, participants rated four items assessing forecasted weekly affect (peaceful, excited, sad, anxious), one per valence/arousal combination, whereas the weekly experienced affect scale included all eight items (two per valence/arousal combination). We re-ran the main weekly forecasting analyses using the same two-item scales for forecasted as experienced positive and negative affect, meaning the forecasted and experienced affect scales were directly comparable. Our findings were unchanged (see Supplementary Table S7).

For daily forecasts, participants forecasted affect for tomorrow in the end-of-day surveys (*daily forecasted affect*) and rated their experienced affect seven times for the past day in each end-of-day survey (*daily retrospective affect*), and nine times daily in each momentary survey (*momentary affect*). Because we had two dynamic measures of experienced affect (momentary, retrospective) but only one dynamic measure of forecasted affect (daily forecasted affect), we prioritised getting an unbiased estimate of forecasted affect. Thus, in each end-of-day survey, participants forecasted tomorrow’s affect *before* rating today’s affect. We statistically controlled for retrospective affect biasing forecasted affect ratings by re-running the daily forecasting models controlling for yesterday’s retrospective affect (also lagged—Supplementary Table S8), which yielded results consistent with our main analyses.

### Data-Analytic Strategy

We used *R* (Version 4.4.2) to run linear regression models with the *lm* package, multilevel linear regression models with the *lme4* package (Bates et al., 2015), and t-tests with the *rstatix* package. We tested statistical significance of model parameters using *lmerTest* (Kuznetsova et al., 2017). We modelled positive and negative affect separately, and ran supplementary analyses on the eight specific emotion items. Because we had both retrospective and momentary experienced affect measures, we ran each model twice, once per timeframe.

We used two- and three-level models. The two-level models accounted for the nesting of days (Level-1) within people (Level-2). The three-level models accounted for the nesting of moments (Level-1) within days (Level-2) within people (Level-3). Because level number varied by model type (two- vs. three-level), we refer to levels as moment-level, day-level, and person-level. We grand-mean centered all person-level (i.e., time invariant) predictors. Unless otherwise specified, we person-mean centered all moment- and day-level (i.e., time varying) predictors to ensure they represented purely within-person effects. Because participants’ daily forecasts were about the next day’s affect, the forecasted affect item was lagged (i.e., from yesterday).

We initially included all possible random intercepts and slopes. To address singular fit issues we removed, one-by-one, the random slopes at each level that most strongly correlated with other random effects. We ensured that the fixed effects stayed consistent across changes to the random effects structure. See the analysis code (https://osf.io/qcksn) for details on the random effects structure for each model.

#### Modelling Forecasting Accuracy

We modelled relative and absolute forecasting accuracy for the next day/week. Because participants made daily forecasts repeatedly, we were able to model within- and between-person effects in the daily forecasting models. In contrast, as participants forecasted their affect for the upcoming week once, we could only test between-person effects in the weekly forecasting models.

The relative accuracy analyses tested whether participants experienced higher affect the next day/week when they forecasted higher affect than usual. In these models, we included both person-mean centered and grand-mean centered forecasted affect. Thus, we simultaneously examined the within-person effect (i.e., do people experience higher affect on days they forecast higher affect than usual) and the between-person effect (i.e., do people who forecast higher affect experience higher affect, relative to other people) of forecasted on experienced affect.

The absolute accuracy analyses (adapted from Neubauer et al., 2020) tested for mean differences in forecasted and experienced affect. Following Neubauer et al., we stacked the forecasted and experienced affect ratings from each day, resulting in a categorical variable of rating-type (forecasted, experienced) paired with a continuous variable for rating level. We then used multilevel models where rating-type (forecasted, experienced) was the categorical predictor, and affect levels the outcome. Because these analyses look at mean differences, we first calculated day-means of momentary affect ratings. The fixed slope of rating-type represents the direction and magnitude of absolute forecasting errors, relative to experienced affect levels (underestimation < 0, perfect accuracy = 0, overestimation > 0). For weekly forecasts, we ran paired sample *t*-tests comparing each participant’s forecast to their (1) mean momentary affect across the week, and (2) weekly retrospective affect rating.

## Results

Table 2 displays descriptive statistics for key variables in Study 1. The ICC values (0.52–0.66) indicate that all repeated measures comprise substantial between- and within-person variance. We report between- and within-person correlations in Supplementary Tables S9-S10.

**Table 2:** Descriptive statistics for key variables in Study 1

	*M*	Within person* SD*	Between person* SD*	ICC
*Forecasted affect*				
Weekly forecasted positive affect	46.73	-	21.15	-
Weekly forecasted negative affect	39.70	-	21.98	-
Daily forecasted positive affect	46.59	11.87	16.20	0.58
Daily forecasted negative affect	29.53	10.10	16.95	0.66
*Experienced affect*				
Weekly retrospective positive affect	48.88	-	18.86	-
Weekly retrospective negative affect	31.60	-	19.78	-
Daily retrospective positive affect	44.56	11.46	16.02	0.60
Daily retrospective negative affect	29.45	11.14	16.56	0.61
Momentary positive affect	43.44	13.26	14.80	0.52
Momentary negative affect	23.72	11.28	14.93	0.59

Scores for all variables range from 0 (not at all) to 100 (very much). ICC refers to intraclass correlation coefficients, where the number indicates the proportion of variance between-person. Forecasted affect refers to predictions people made for how they would feel tomorrow and over the next week. Experienced affect refers to how people actually felt

### Are People Accurate At Predicting How They Will Feel Tomorrow and Next Week?

#### Daily Forecasting Accuracy

In terms of relative accuracy (see Table 3), as hypothesized, participants’ daily forecasted affect significantly positively predicted their experienced affect the next day, both for momentary and daily retrospective affect ratings. Similarly, people who forecasted higher affect levels experienced higher affect levels. These findings were consistent across positive and negative affect and for specific emotions^4^ (e.g., sad, enthusiastic; Supplementary Table S11). Thus, when participants expected to feel more positive/negative affect than usual tomorrow, they reported experiencing significantly higher-than-average levels of positive/negative affect the following day, showing relative forecasting accuracy.^5^

**Table 3:** Multilevel model estimates: relative accuracy of daily affective forecasts

	Momentary Experienced Affect			Retrospective Experienced Affect		
	Estimate (*SE*)	95% CI	*p*	Estimate (*SE*)	95% CI	*p*
*Positive affect*						
Intercept	43.36 (0.41)	42.55–44.18	< 0.001	44.42 (0.44)	43.55–45.30	< 0.001
Daily forecasted affect (person-mean centered)	**0.26** **(0.02)**	**0.22–0.31**	**< 0.001**	**0.31 (0.03)**	**0.25–0.37**	**< 0.001**
Person mean daily forecasted affect (grand-mean centered)	**0.83 (0.03)**	**0.78–0.89**	**< 0.001**	**0.93 (0.03)**	**0.87–0.98**	**< 0.001**
*Negative affect*						
Intercept	23.67 (0.45)	22.78–24.55	< 0.001	29.30 (0.48)	28.08–29.97	< 0.001
Daily forecasted affect (person-mean centered)	**0.25** **(0.03)**	**0.19–0.30**	**< 0.001**	**0.27 (0.04)**	**0.19–0.35**	**< 0.001**
Person mean daily forecasted affect (grand-mean centered)	**0.79** **(0.03)**	**0.73–0.84**	**< 0.001**	**0.92 (0.03)**	**0.86–0.97**	**< 0.001**

Note. Participants rated momentary experienced affect nine times per day. Each evening, participants rated retrospective experienced affect for that day. Bold indicates significant fixed-effects for predictors of interest

**Fig. 1:**
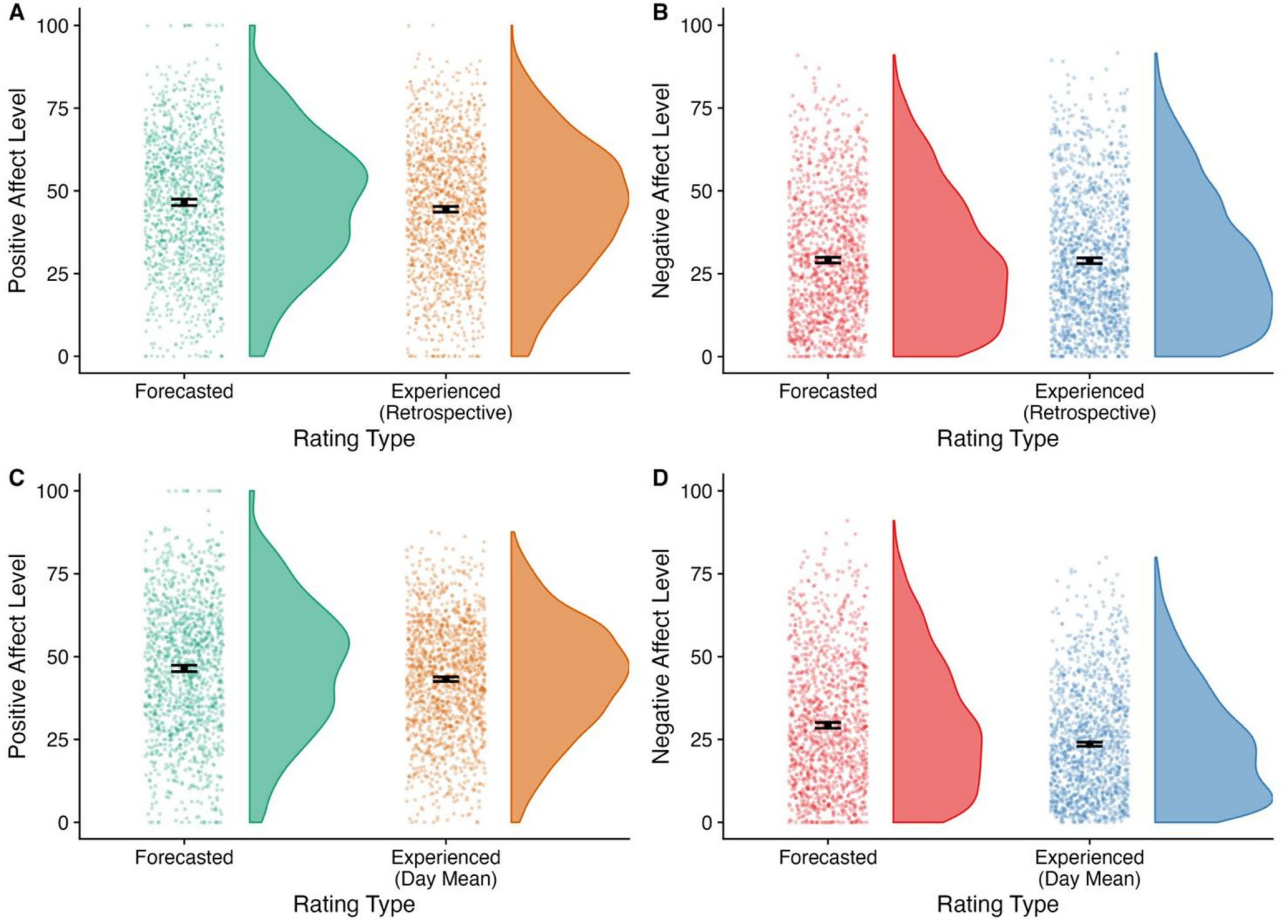
Absolute accuracy of daily forecasts of positive and negative affect.* Note*: The scatterplot represents the raw data, and the rain plot represents the data spread. The black dot and error bar represent the mean, with a 95% CI. Participants slightly overestimated daily affect (*p* <.001), except when we compared forecasted negative affect to daily retrospective negative affect (Fig. 1B)

Turning to absolute accuracy of daily forecasts, participants slightly overestimated their daily positive affect level, as demonstrated by a statistically significant *positive* slope of rating-type when comparing forecasted affect to both daily retrospective affect (Est.=2.07, *SE* = 0.48, 95% CI [1.13, 3.01], *p*<.001; Fig. 1A) and the day-mean of momentary affect ratings (Est.=3.27, *SE* = 0.44, 95% CI [−2.42, 4.13], *p*<.001; Fig. 1C). These effects were small, given the potential range from + 100 (maximum overestimation) to −100 (maximum underestimation). For the supplementary analyses with specific emotions, we similarly found overestimation for the high arousal positive emotions (excited, enthusiastic). However, participants tended to accurately predict, and in one analysis underestimated, the low arousal positive emotions (relaxed, peaceful; see Supplementary Table S12).

Participants also overestimated negative affect, but only when we compared forecasted affect to the day-mean of momentary negative affect ratings (Est.=5.73, *SE* = 0.40, 95% CI [4.95, 6.52], *p*<.001; Fig. 1D). This finding replicated for each specific negative emotion (see Supplementary Table S12). In contrast, there was no significant effect of rating-type when comparing forecasted negative affect to daily *retrospective* negative affect ratings (Est.=−0.19, *SE* = 0.46, 95% CI [−1.09, 0.71], *p* =.684; Fig. 1B). We similarly found no significant difference in the specific emotion supplementary analyses, except for anxiety, where we found overestimation (see Supplementary Table S13).

#### Weekly Forecasting Accuracy

For relative accuracy, affective forecasts significantly predicted momentary affect aggregated across the week (see Table 4), suggesting people who forecasted higher affect experienced higher affect. However, weekly forecasted affect did not significantly predict retrospective ratings of experienced affect made at the end of the week, perhaps due to fewer observations in these models (the result of a programming error in sending the experience-sampling surveys; *N =* 157). For absolute accuracy (see Table 2 for descriptive statistics), forecasted positive affect did not significantly differ from weekly retrospective positive affect (*M*_diff=_−2.15), *t*(156) = 1.08, *p*=.28, *d =* 0.09. However, participants slightly overestimated their positive affect for the upcoming week, relative to the aggregate of momentary positive affect ratings across the week (*M*_diff=_3.29), *t*(209)= −2.88, *p*=.004, *d =* 0.20. We found evidence of absolute overestimation in forecasting negative affect for the upcoming week: participants’ forecasted negative affect was higher than both their weekly retrospective (*M*_diff=_8.10, *t*(157)=−3.85, *p*<.001, *d =* 0.31) and their aggregated momentary (*M*_diff=_15.98, *t*(209)= −12.90, *p*<.001, *d =* 0.89) negative affect.

**Table 4:** Model estimates: relative accuracy of weekly affective forecasts

	Momentary Experienced Affect			Weekly Retrospective Experienced Affect		
	Estimate (*SE*)	95% CI	*p*	Estimate (*SE*)	95% CI	*p*
*Positive affect*						
Intercept	43.39 (0.81)	41.78–44.99	< 0.001	48.89 (1.50)	45.93–51.85	< 0.001
Weekly Forecasted Affect	**0.43 (0.04)**	**0.35–0.51**	**< 0.001**	0.11 (0.07)	−0.03–0.26	0.125
*Negative affect*						
Intercept	23.76 (0.84)	22.12–25.41	< 0.001	31.61 (1.59)	28.48–34.75	< 0.001
Weekly Forecasted Affect	**0.40 (0.04)**	**0.33–0.48**	**< 0.001**	−0.01 (0.07)	−0.15–0.13	0.884

The models with momentary experienced affect as the outcome were multilevel models. The models with weekly retrospective affect as the outcome were not multilevel models. Bold indicates significant fixed-effects for predictors of interest

## Discussion

In Study 1, we found that (1) participants who forecasted higher positive/negative affect overall experienced higher positive/negative affect and (2) participants were able to accurately predict whether they would feel more positive/negative relative to a typical day. But participants tended to slightly overestimate *absolute* levels of both positive and negative affect, especially when comparing forecasted affect to their experienced affect ratings made in the moment. These findings are consistent with Wirtz et al. (2003) who found that participants overestimated both positive and negative affect experienced during a lengthy vacation.

While Study 1 sheds light on the accuracy of everyday affective forecasts over relatively short time-periods, it leaves two key gaps. First, contrary to most previous forecasting research, we focused on forecasts of general affect, rather than forecasts for specific events. By asking participants to predict how they would feel over an entire day or week, we cannot determine what information people were drawing on to make their forecasts. We assume that participants’ affective forecasts for the next day or week were likely informed by their predictions about what would happen on a day-to-day basis. However, the extent to which participants’ affective forecasts were informed by one specific situation occurring the next day or the next week, multiple situations, or their general anticipated mood is unknown.

Second, we assessed participants’ next-day forecasted affect each evening rather than asking participants to predict how they would feel on the same day each morning. We used this approach to prevent people acting in accordance with their forecasts. But consequently, we could not control for changes in forecasted affect due to sleep quality. Given that sleep quality is closely related to next-day affect (Ten Brink et al., 2022), affective forecasts for a given day may be more accurate when made in the morning, when people can incorporate the influence of their previous night’s sleep. We addressed these gaps in Study 2, in which participants made forecasts each morning about their affective responses to an unpleasant event expected to occur the same day, and then rated their experienced affect for the same event each evening.

### Study 2: Affective Forecasting for Everyday Unpleasant Events

We sought to determine the accuracy of forecasts for everyday unpleasant events by analysing data from a two-week daily diary study. For relative accuracy, our registered hypothesis was that forecasted positive and negative affect would positively predict experienced positive and negative affect, replicating our findings in Study 1. In contrast, we did not expect to replicate our Study 1 findings for absolute accuracy given that Study 2 focussed exclusively on forecasting affective responses to unpleasant events rather than predicting general daily affect. We instead derived our hypotheses from Buechel et al.’s (2017) theory.

Buechel et al. (2017) propose that the (1) expected impact, (2) probability, and (3) psychological distance of events will predict whether people will overestimate or understate their emotional responses to events. People will be most likely to overestimate their emotions for future events that are expected to have a large impact, are highly probable, and psychologically close—in the direction that aligns with the hedonic impact of the event (i.e., overestimate negative affect for unpleasant events, and positive affect for positive events).

For Study 2, participants made forecasts about the event they were least looking forward to that day. These events can be classified as psychologically near and quite probable, but their expected impact likely varies depending on specific event details. Buechel et al. (2017) propose that these three outcome specifications work additively to predict affective forecasts. Thus, given that the events in Study 2 likely satisfy two out of three of Buechel et al.’s criteria, we predicted that participants would overestimate event-related negative affect (forecasted negative affect ratings will be higher than experienced negative affect ratings) and underestimate event-related positive affect (forecasted positive affect ratings will be lower than experienced positive affect ratings). Because we focus on everyday events rather than significant rare events, we expected small forecasting errors. Based on the size of the absolute accuracy errors in Study 1, we defined “small” as less than 5% (i.e., fewer than 0.25 scale points on the 1–5 scale that participants made affect ratings on in Study 2).

## Method

### Transparency and Openness

We conducted secondary analyses on a daily diary dataset collected to answer other research questions (e.g., Mehta et al., 2025). We post-registered our hypotheses and analysis plan (https://osf.io/rtb3x) before analysing the data. See https://osf.io/u5htx for data and https://osf.io/xvb47 for analysis code. Below, we focus on details relevant to the current study.

### Participants

The original research team determined sample size based on the number of participants they could recruit in one academic quarter. While 103 participants completed the baseline survey, only those who passed all three attention checks embedded in the baseline survey (e.g., *“I respond to surveys randomly. Choose strongly disagree”*) were invited to the daily diary portion of the study (*N* = 73). Of these participants, 69 completed at least one daily diary survey.

The final sample (*N* = 69) comprised local college students and Stanford University alumni aged 18–77 years (*M* = 33.7, *SD* = 17.1 years).^6^ University participants received course credit for participating, while alumni participants volunteered their time. The sample was mostly women (79.7%; 51 women, 13 men). Participants identified as Asian or Asian American (34.4%), White/Caucasion (28.1%), Latino/Hispanic (25%), Multiracial or Multiethnic (6.25%), Black or African American (1.56%), Pacific Islander (1.56%), and Other or Declined to answer (3.12%).

### Procedure

The study ran for 15 consecutive days and comprised a baseline survey (Day 0), morning and evening daily diary surveys (Days 1–14), and an exit survey (Day 14). Neither the baseline nor exit surveys are relevant to the current study and are therefore not described further. Before starting the daily diaries, participants completed a brief online training that included videos explaining the daily-diary procedure, some walk-through practice questions, and knowledge checks.

The daily diaries followed a fixed-sampling scheme. Participants were sent a link to the morning survey at 05:00am and the evening survey at 8:30pm each day. They completed each survey via Qualtrics, with links sent via Telegram using the application EMA Ping Bot (https://emapingbot.com/). Participants were instructed to complete the morning survey shortly after waking up and the evening survey shortly before going to sleep. There were 732 completed morning surveys (compliance: *M* = 85.6%; *median* = 92.9%, *SD* = 18.1%) and 703 completed evening surveys (compliance: *M* = 84.7%, *median* = 92.9%, *SD* = 18.7%). We did not exclude any survey items, or observations, based on completion time.

#### Morning Survey

In the morning survey, participants nominated their target unpleasant situation for that day. They were instructed: *“Thinking ahead about your day*,* please think of the one thing that you are least looking forward to*,* i.e. that you expect to be most unpleasant.”* Participants then briefly described this situation, before rating how negative and positive they expected the situation to make them feel at its most negative/positive point (*forecasted peak intensity;* see Supplemetary Materials for details and Supplementary Table S14 for results). Participants then rated how they expected the situation to make them feel on specific affect items (*event-related forecasted affect)*.

#### Evening Survey

In the evening survey, participants were shown the event description they provided in the morning and asked “*Did the situation go as you expected when you wrote about it this morning?”* (0 = not at all, 1 = slightly, 2 = moderately, 3 = very much). Participants then described whether anything unexpected happened during the situation, or whether the situation did not occur. There were 176 surveys where participants indicated the situation did not go at all as they expected (i.e., a rating of 0). We inspected the descriptions accompanying these ratings and excluded 28 observations where it was clear the situation did not occur, as participants cannot rate their experienced affect for events that did not occur. Participants then rated how negative/positive the situation made them feel at its most negative/positive point (*experienced peak intensity;* see Supplementary Materials) followed by rating the specific affect items (*event-related experienced affect*).

### Measures

#### Positive and Negative Affect

There were two positive^7^ (low-arousal: *relaxation;* high-arousal: *happy*) and six negative (low-arousal: *sadness*,* boredom*,* embarrassment or shame*; high-arousal: *anxiety or fear*, *disgust*,* anger*) affect items. In the morning survey, participants rated each affect item in relation to how they expected their unpleasant situation to make them feel (e.g., *During this situation*,* I expect I will feel sadness*) from 1 (do not expect to feel this way at all) to 5 (extremely expect to feel this way). In the evening survey, participants rated the same affect items as the morning survey, but in relation to how the situation made them feel (e.g., *“Looking back at my day*,* this situation made me feel sadness”*). The forecasted and experienced positive and negative affect scales were reliable within- and between-person (see Table 5).

**Table 5:** Descriptive statistics for key variables in Study 2

Variable	*M*	Within person *SD*	Between person *SD*	ICC
Forecasted positive affect	1.59	0.60	0.54	0.32
Forecasted negative affect	1.86	0.47	0.48	0.39
Experienced positive affect	1.76	0.75	0.66	0.30
Experienced negative affect	1.71	0.47	0.47	0.35

Note. Scores for affect ratings range from 1 (not at all) to 5 (extremely). Forecasted affect refers to predictions people made for how they expected the unpleasant situation to make them feel that day. Experienced affect refers to how people actually felt once the situation occurred

### Data Analytic Strategy

We ran two-level models to account for days being nested within people. We used the same packages and overall modelling approach (including for the random effects structure) as Study 1. We first tested relative accuracy and then absolute accuracy. Our primary interest was in forecasting accuracy for positive and negative affect, but we ran supplementary models with forecasted and experienced positive and negative peak intensity (see Supplementary Table S14). We selected the affect items as our primary interest because the affect items were more similar to the Study 1 items than the peak intensity items. However, we found the same results in the intensity models as our main affect models. As in Study 1, we also ran supplementary analyses on the eight specific affect items (see Supplementary Tables S15-S16).

## Results

Table 5 displays descriptive statistics. In general, participants forecasted and experienced their affective responses to unpleasant events as mildly negative and mildly positive. Intraclass coefficient (ICC) values between 0.30 and 0.39 indicate that the variance for all variables was predominantly within persons. We report between- and within-person correlations for Study 2 in Supplementary Table S17.

### Are People Accurate At Predicting How Unpleasant Everyday Events Will Make Them Feel?

Regarding relative forecasting accuracy, as in Study 1, we found positive associations between forecasted and experienced positive and negative affect at both the within- and between-person level (Table 6). On days that participants expected to feel more positive/negative than usual about their upcoming unpleasant event, they experienced higher than average levels of positive/negative affect in response to these events. Similarly, people who tended to forecast higher positive/negative affect experienced higher levels of positive/negative affect. These positive associations for relative accuracy replicated for all eight specific emotion items (Supplementary Table S15). Thus, participants showed relative forecasting accuracy for event-related affect.

**Table 6:** Multilevel model estimates: relative accuracy of event-related affective forecasts

	Experienced positive affect			Experienced negative affect		
	Estimate (*SE*)	95% CI	*p*	Estimate (*SE*)	95% CI	*p*
Intercept	1.80 (0.05)	1.70–1.90	< 0.001	1.67 (0.03)	1.61–1.73	< 0.001
Forecasted affect (person mean centered)	**0.61** **(0.06)**	**0.49–0.73**	**< 0.001**	**0.48** **(0.05)**	**0.38–0.57**	**< 0.001**
Person mean forecasted affect (grand mean centered)	**0.34** **(0.10)**	**0.14–0.55**	**0.001**	**0.27** **(0.07)**	**0.14–0.40**	**< 0.001**

Bold indicates significant fixed-effects for predictors of interest

**Fig. 2:**
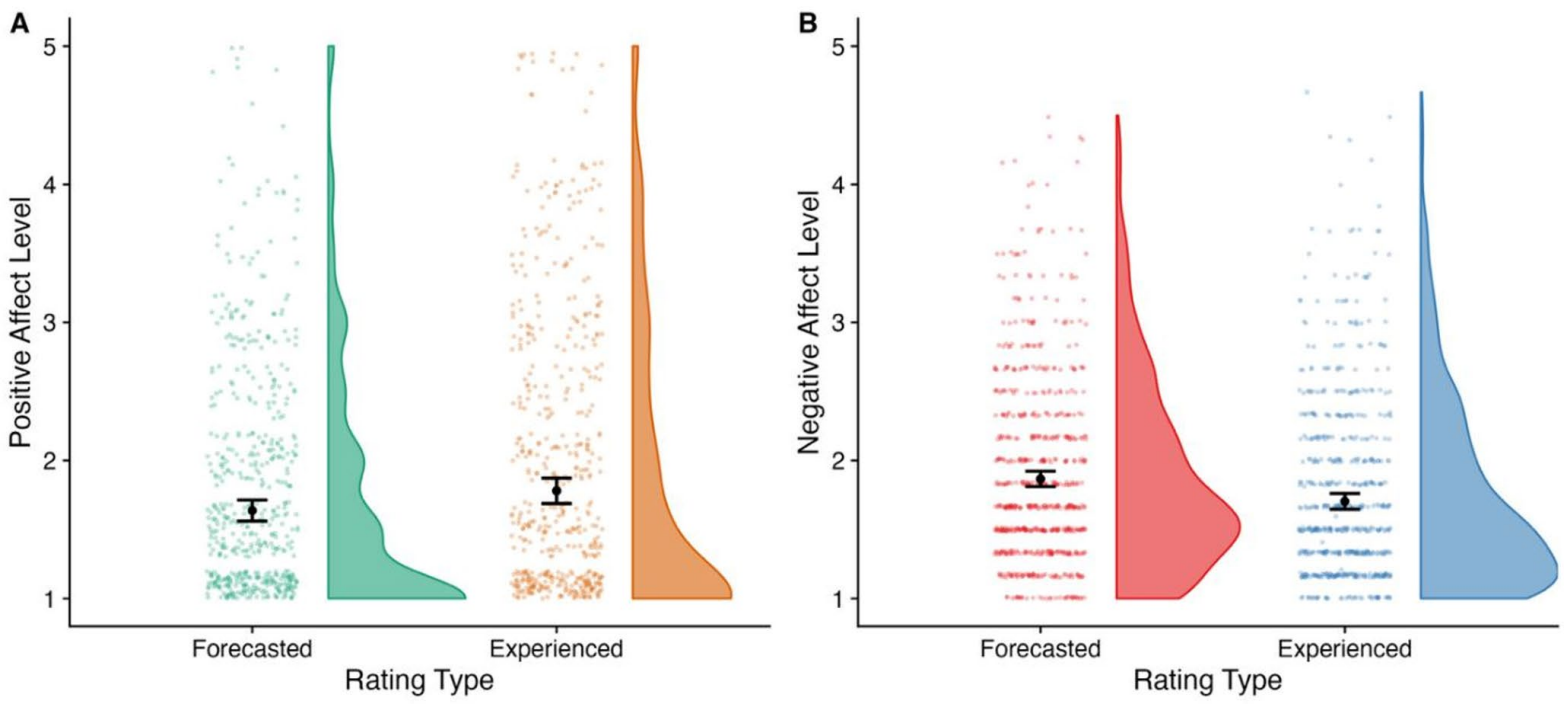
Absolute Accuracy of Unpleasant Event-Related Forecasts of Positive and Negative Affect. Note. The scatterplot represents the raw data, and the rain plot represents the data spread. The black dot and error bar represent the mean, with a 95% CI. Participants underestimated positive affect and overestimated negative affect in relation to daily unpleasant events

Turning to absolute accuracy, we found evidence of small forecasting errors in the hypothesized directions, which replicated in supplementary analyses using participants’ ratings of peak affect intensity (see Supplementary Table S14). Participants underestimated how positive they would feel in response to their unpleasant event. This effect was demonstrated by a statistically significant negative slope of rating-type (Est.=−0.15, *SE* = 0.04, 95% CI [−0.23, −0.06], *p*=.001; Fig. 2A). Thus, compared with experienced positive affect—represented by the model intercept (Est.=1.76, *SE* = 0.07, 95%CI [1.61, 1.90], *p*<.001)—participants forecasted lower positive affect in response to their daily unpleasant events. The supplementary analyses for specific emotion items showed that this tendency to underestimate occurred for both happiness and relaxation (Supplementary Table S16).

In contrast, participants tended to overestimate how negative they would feel in response to their unpleasant event, as shown by a statistically significant positive slope of rating-type (Est.=0.16, *SE* = 0.03, 95% CI [0.11, 0.22], *p*<.001; Fig. 2B). Thus, compared with participants’ ratings of experienced negative affect—represented by the intercept (Est.=1.71, *SE* = 0.06, 95%CI [1.60, 1.82], *p*<.001)—participants forecasted their negative affect to be higher.

However, supplementary analyses suggested the tendency to overestimate varied across different negative affect items. Similar to the overall negative affect findings, participants tended to overestimate how bored, embarrassed, anxious, and angry they would feel in relation to their unpleasant event. Notably, the estimates for boredom and anxiety were double the size of the estimate for overall negative affect. However, for sadness and disgust, we found no signficant difference in forecasted and experienced affect levels. Together, these findings suggest that overall, participants made small absolute forecasting errors in directions consistent with the hedonic valence of the event. But when we look at specific affect items, people seem to be more accurate in forecasting some negative emotions (e.g., disgust, sadness) than others (boredom, embarrassment, anxiety/fear, anger) in relation to unpleasant events.

## General Discussion

People often base decisions on predictions about how they will feel in the future. Everyday decision-making likely involves forecasting affective responses to everyday events or over relatively short time periods such as one day. Yet, previous research has predominantly focussed on affective forecasts for rare, rather than everyday, events (Patel & Urry, 2024). We addressed this gap in two intensive longitudinal studies: an experience sampling study focused on daily and weekly forecasts, and a daily diary study focused on forecasts for everyday unpleasant events. Participants showed relative forecasting accuracy, as they were consistently able to identify when a day or specific event would make them feel more or less positive/negative than usual. Although participants were sometimes inaccurate in absolute terms, when errors occurred, they were small. Together, these findings show that people are reasonably accurate at affective forecasting in everyday life.

The direction of errors when forecasting absolute affect levels varied by whether the forecasts were about a specific time period (Study 1) or a specific event (Study 2). When participants forecasted how they would feel tomorrow or over the next week, they tended to slightly overestimate *both* their positive and negative affect levels (Study 1). These findings are consistent with other experience-sampling studies showing people overestimate affect for the upcoming week in absolute terms (Mathersul & Ruscio, 2020; Wenze et al., 2012), though contrast with Colombo et al.’s (2020) findings that people underestimated their future positive and negative feelings over a two-week period.

When forecasting how they would feel about a specific unpleasant event occurring that day (Study 2), however, participants overestimated how negative but underestimated how positive they would feel in response to the event. The tendency to overestimate absolute levels of negative affect for unpleasant events is consistent with many previous affective forecasting studies (for reviews, see Bosch et al., 2021; Mathieu & Gosling, 2012). The tendency to underestimate positive affect for events assumed to be negative is consistent with research showing people underestimate positive responses to interacting with strangers (e.g., Epley & Schroeder, 2014).

Together, our Study 2 findings suggest—consistent with Buechel et al.’s (2017) theory—that when people think about specific events that have a clear valence, the direction of forecasting errors is tied to the hedonic valence of the event. Whereas, the effects from Study 1, alongside prior work, suggest that when people think about broad time periods (Mathersul & Ruscio, 2020; Wenze et al., 2012) or lengthy situations (e.g., vacations; Wirtz et al., 2003) that encompass both pleasant and unpleasant events as well as general mood, they tend to overestimate *both* positive and negative affect in absolute terms. Thus, the target of affective forecasts (e.g., broad time-periods vs. specific situations) matters for whether people overestimate affect in general or show a valence-dependent pattern of forecasting errors.

Our findings show that whether experienced affect is measured in the moment, or retrospectively, can change the “accuracy” of affective forecasts. In Study 1, we found that the size of absolute overestimation errors—and for negative affect, whether overestimation occurred at all—varied by which experienced affect rating the forecast was compared with. The tendency to overestimate was larger when we compared forecasted affect to (mean) momentary ratings, than to retrospective end-of-day affect ratings. This pattern has been observed in other research, where forecasted and remembered emotions about a vacation were both higher than mean momentary emotions made during the same period (Wirtz et al., 2003). The closer match between forecasted and retrospective ratings may be due to both forecasts and retrospections drawing on memory—which is known to be biased—while momentary ratings draw on experiential knowledge (Robinson & Clore, 2002). Researchers should consider how the type of experienced affect measure (momentary vs. retrospective) might be influencing their estimates of forecasting accuracy. If the intention is to determine how forecasted affect compares to affective experience during a specific moment or event (e.g., the commute to work), then researchers should assess feelings during that focal event or time-period in the moment, rather than retrospectively.

While our main focus was on investigating forecasting accuracy for positive and negative affect overall, in supplementary analyses we investigated forecasting accuracy for specific emotions. Emotion type made no difference to relative accuracy, but mattered for some estimates of absolute accuracy. When forecasting tomorrow’s affect (Study 1), the type of positive emotion mattered: participants overestimated high-arousal positive emotions (excited, enthusiastic), but were accurate or underestimated low-arousal positive emotions (relaxed, peaceful). There was some, though less consistent, evidence that arousal mattered for the accuracy of forecasted negative emotions: participants overestimated anxiety (a high arousal negative emotion) when they were accurate in forecasting negative affect overall. However, for forecasts of daily unpleasant events (Study 2), the specific emotion findings mapped less clearly to arousal distinctions. These findings tentatively suggest arousal as a factor relevant to understanding affective forecasting accuracy.

These results should be interpreted in light of some limitations. First, Study 2 had a small person-level sample size. Though our findings replicated across peak intensity and retrospective affect ratings, it would be worth replicating these findings in a larger sample, similar in size to Study 1. Second, in Study 2 there was mismatched wording between the forecasted and experienced affect items. Participants were asked to “look back at their day” before rating their experienced affect, which may have promoted a broader view of the situation than if they had been asked to only consider the situation itself. Relatedly, before rating their experienced affect in the evening survey, participants indicated whether the situation nominated in the morning survey went as they expected. The order of these items was not counterbalanced. We therefore cannot exclude that asking participants if the situation went as expected influenced their experienced affect ratings. Third, in Study 2 we only focused on unpleasant everyday events and did not ask about pleasant events, meaning we have an incomplete picture of what affective forecasting for everyday events looks like. Addressing this limitation is needed to fully test Buechel et al.’s (2017) theory that when making forecasts for specific events, the direction of forecasting errors is consistent with the hedonic valence of the event. Future research should investigate pleasant and unpleasant everyday events to confirm the reverse pattern occurs for hedonically positive events: that people overestimate positive and underestimate negative affect.

People often think about how they will feel in the future. Here, we showed that in everyday life, these predictions reflect subsequent feelings in relative terms, but that people sometimes make small errors in absolute terms—consistent with affective forecasting research on more rare events. People tend to slightly overestimate how positive and negative they think they will feel *tomorrow* and *next week*. Whereas people tend to slightly overestimate how negative, but underestimate how positive, *everyday unpleasant events* will be. This research lays the groundwork for future work identifying the psychological function of making small absolute forecasting errors in everyday life.

## Supplementary Information

Below is the link to the electronic supplementary material.

Supplementary Material 1 (DOCX 894 KB)

## Footnotes

1 We use the term affect, rather than emotion for two reasons: (1) to match the term *affective* forecasting, and (2) because Study 1 focuses on general, not event-related, feelings (Gross & Thompson, 2007).

2 Some analyses use only end-of-day survey data, with a cluster size of 7. However, since power in multilevel designs is more strongly determined by the number of upper-level units (here, participants) than by cluster size (Bolger & Laurenceau, 2013), we do not anticipate substantial losses in power for the end-of-day analyses.

3 Only 157 participants completed the Day-7 end-of-day survey, due to a programming technical error.

4 To reduce the number of models, we analysed relative accuracy in forecasting specific emotions using the retrospective experienced affect ratings only (not momentary).

5 These findings replicated when we included yesterday’s experienced affect as a control variable (Supplementary Table S8).

6 We are missing baseline data from 5 participants and therefore report demographics for *N* = 64.

7
*Desire or longing* was also included as a positive affect item. However, as specified in our post-registration, given that the reliability of the positive affect scale was higher without this item, we used the two-item positive affect scale (relaxation, happy) for our analyses.

## Additional Information

### Availability of Data and Material

We pre-registered the broader data collection procedure for Study 1 [https://osf.io/rwbgj/] on the Open Science Framework. We separately registered our analysis plan for both studies after data were collected but before they were analyzed [Study 1: https://osf.io/86fgv/, Study 2: https://osf.io/rtb3x]. Data available at https://osf.io/tgb4x (Study 1), https://osf.io/u5htx (Study 2). Analysis code available at https://osf.io/qcksn (Study 1), https://osf.io/xvb47 (Study 2).
