# Supplementary material for: Affective Forecasting Accuracy in Everyday Life
Source: Affect Sci. 2026 Mar 7;7(2):306–18. doi: 10.1007/s42761-026-00364-x (PMC13269588; doi:10.1007/s42761-026-00364-x)
Supplement: Supplementary file 1 — Supplementary Material 1 (DOCX 894 KB) [file 42761_2026_364_MOESM1_ESM.docx]

**Supplementary Material for Moeck et al. “Affective Forecasting Accuracy in Everyday Life”**

Table of Contents

[Additional pre-registered analyses for Study 1: investigating whether forecasting accuracy is associated with emotional benefits 3](#_Toc217049451)

[Measures 3](#_Toc217049452)

[Figure S1 6](#_Toc217049453)

[Example Response Surface Supporting a Congruence Hypothesis. The Outcome Variable was Either Emotion-Focused Coping or Daily Life Satisfaction. 6](#_Toc217049454)

[Results 6](#_Toc217049455)

[Table S1 8](#_Toc217049456)

[Multilevel Regression Model Estimates: Association Between Forecasting Accuracy and Emotion-Focused Coping and Daily Life Satisfaction 8](#_Toc217049457)

[Table S2 9](#_Toc217049458)

[Results of the Regression Models Testing Forecasting Accuracy Consequences Including Yesterday’s Affect as a Control Variable 9](#_Toc217049459)

[Figure S2 10](#_Toc217049460)

[Response Surfaces for Multilevel RSA Testing Whether Congruence Between Forecasted and Experienced Positive Affect (PA) or Negative Affect (NA) Predicted Emotion-Focused Coping (Panels A, C, E, G) or Daily Life Satisfaction (Panels B, D, F, H) 10](#_Toc217049461)

[Full Results of the Response Surface Analyses Testing Whether Forecasting Accuracy Confers Emotional Benefits 11](#_Toc217049462)

[Table S3 11](#_Toc217049463)

[Response surface and multilevel polynomial coefficients for the models testing whether congruence between forecasted and experienced (day mean) positive affect predicts emotion-focused coping or daily life satisfaction. 11](#_Toc217049464)

[Table S4 12](#_Toc217049465)

[Response surface and multilevel polynomial coefficients for the models testing whether congruence between forecasted and experienced (retrospective) positive affect predicts emotion-focused coping or daily life satisfaction. 12](#_Toc217049466)

[Table S5 13](#_Toc217049467)

[Response surface and multilevel polynomial coefficients for the models testing whether congruence between forecasted and experienced (day mean) negative affect predicts emotion-focused coping or daily life satisfaction. 13](#_Toc217049468)

[Table S6 14](#_Toc217049469)

[Response surface and multilevel polynomial coefficients for the models testing whether congruence between forecasted and experienced (retrospective) negative affect predicts emotion-focused coping or daily life satisfaction. 14](#_Toc217049470)

[Supplementary Forecasting Accuracy Analyses – Study 1 15](#_Toc217049471)

[Table S7 15](#_Toc217049472)

[Model Estimates: Relative Accuracy of Weekly Affective Forecasts – 2 item scales 15](#_Toc217049473)

[Table S8 16](#_Toc217049474)

[Results of the Daily Forecasting Accuracy Models Including Yesterday’s Affect as a Control Variable 16](#_Toc217049475)

[Between and within-person correlations for key Study 1 variables 17](#_Toc217049476)

[Table S9 17](#_Toc217049477)

[Between-person Correlations (r, [95% CI]) Between Weekly Forecasted, Weekly Experienced Affect, and All Other Variables 17](#_Toc217049478)

[Table S10 18](#_Toc217049479)

[Within and Between Person Correlations (r, [95%CI]) For Day- and Momentary-Level Variables 18](#_Toc217049480)

[Additional Supplementary Forecasting Accuracy Analyses – Study 1 19](#_Toc217049481)

[Table S11 19](#_Toc217049482)

[Models Estimating Relative Accuracy in Forecasting Specific Emotions – Study 1: Retrospective Experienced Affect Ratings 19](#_Toc217049483)

[Table S12 20](#_Toc217049484)

[Models Estimating Absolute Accuracy in Forecasting Specific Emotions – Study 1: Day Mean Momentary Affect Ratings 20](#_Toc217049485)

[Table S13 21](#_Toc217049486)

[Models Estimating Absolute Accuracy in Forecasting Specific Emotions – Study 1: Retrospective Experienced Affect Ratings 21](#_Toc217049487)

[Event-related peak positive and negative intensity ratings – Study 2 22](#_Toc217049488)

[Supplementary Forecasting Accuracy Analyses – Study 2 22](#_Toc217049489)

[Table S14 22](#_Toc217049490)

[Multilevel Model Estimates: Relative Accuracy of Event-Related Forecasts of Peak Intensity 22](#_Toc217049491)

[Table S15 23](#_Toc217049492)

[Models Estimating Relative Accuracy in Forecasting Specific Emotions for Unpleasant Events – Study 2 23](#_Toc217049493)

[Table S16 24](#_Toc217049494)

[Models Estimating Absolute Accuracy in Forecasting Specific Emotions for Unpleasant Events – Study 2 24](#_Toc217049495)

[Between and within-person correlations for key Study 2 variables 25](#_Toc217049496)

[Table S17 25](#_Toc217049497)

[Within and Between Person Correlations (r, [95%CI]) For Study 2 Variables 25](#_Toc217049498)

[References 26](#_Toc217049499)

# Additional pre-registered analyses for Study 1: investigating whether forecasting accuracy is associated with emotional benefits

To date, the association between forecasting accuracy and well-being has only been examined at the between-person level, where the tendency to inaccurately forecast may simply be a proxy for dispositional optimism or pessimism (Carver & Scheier, 2014). An additional aim of Study 1 was therefore to test the within-person association between forecasting accuracy and emotion-focused coping as well as daily life satisfaction. Our pre-registered hypothesis was that accurate affective forecasting would be associated with greater emotion-focused coping and daily life satisfaction. We made this hypothesis based on the stress anticipation literature, which suggests that accurate forecasts may help people emotionally prepare for upcoming experiences (e.g., Neupert et al., 2019), while inaccurate forecasts—both by overestimating (Neupert & Bellingtier, 2019) or underestimating (Wang et al., 2020)—may worsen stress.

## Measures

***Emotion-focused coping and daily life satisfaction.***

We included two measures of possible emotional benefits of forecasting accuracy: emotion-focused coping and daily life satisfaction. We assessed emotion-focused coping daily (*“Overall, I have felt able to cope with my emotions today*) and in the last hour (*“In the last hour, I have felt able to cope with my emotions”*; adapted from Erbas et al. (2021). We assessed life satisfaction once daily (*“Overall, how satisfied were you with your life today?”*) because it fluctuates less than affect (e.g., Heller et al., 2006). Participants rated these items from 0 (*not at all*) to 100 (*very much*).

***Modelling whether forecasting accuracy has emotional benefits***

We assessed whether *daily* forecasting accuracy predicted emotion-focused coping or daily life satisfaction in two ways. We first ran multilevel regressions, with forecasting accuracy represented by the interaction between forecasted and experienced affect. These models therefore test the effect of forecasted x experienced affect (i.e., forecasting accuracy) on emotion-focused coping and daily life satisfaction, while controlling for how people felt that day.

To fix convergence issues, we rescaled predictors by subtracting the grand mean and dividing by the grand *SD*. Because the variables were person-mean centred *before* rescaling, this procedure did not change within-person effects interpretation. To aid interpretation, we created the tables and figures on the unscaled models. To address singular fit issues we removed, one-by-one, the random slopes at each level that most strongly correlated with other random effects. In models including interactions between two predictors, we always removed the random slope for the interaction term before removing any main effect slopes. We ensured that the fixed effects stayed consistent across changes to the random effects structure. See the analysis code (<https://osf.io/qcksn>) for details on the random effects structure for each model.

As a more robust and comprehensive test of the emotional benefits of forecasting accuracy, we also ran multilevel response surface analyses (RSA; Nestler et al., 2019), which use effects from a multilevel polynomial regression to generate a 3D response surface. RSA tests whether the congruence between two variables (i.e., forecasted and experienced affect) predicts higher levels of an outcome (i.e., coping/life-satisfaction), while avoiding limitations of difference scores (e.g., masking information about affect level; Le et al., 2020) and moderated regression (e.g., comparing arbitrary predictor levels). Congruence is shown by a response surface highest at the maximum values of the two predictors, with a similarly steep slope down to the minimum values of the two predictors (see Figure S1).

Consistent with Nestler et al. (2019), we person mean centered the predictors (and their squared and interaction terms) around the pooled mean of both predictors. Multilevel RSA require the two predictor variables to be on the same level (Nestler et al., 2019). Therefore, for models investigating the match between forecasted and *momentary* experienced affect, we aggregated momentary affect ratings to the day-level and only used daily, not momentary, coping ratings. We ran eight models: for each outcome (emotion-focused coping, life satisfaction), there were two positive and two negative affect models, one per experienced affect operationalisation (momentary day-mean, daily retrospective).

## Figure S1

### Example Response Surface Supporting a Congruence Hypothesis. The Outcome Variable was Either Emotion-Focused Coping or Daily Life Satisfaction.

###


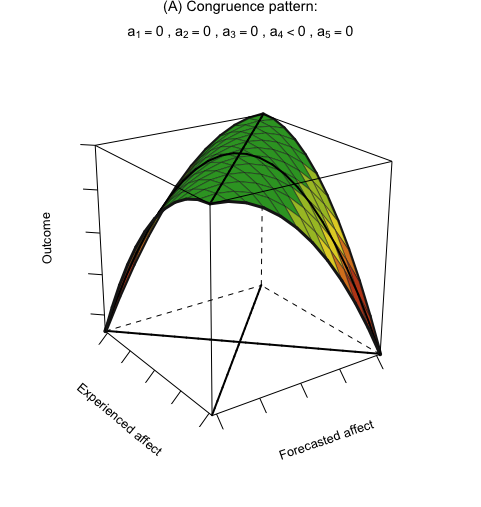


## Results

Participants reported high levels of emotion-focused coping (momentary: *M* = 74.85, *SD_within_* = 15.31, *SD_between_* = 18.18; daily: *M* = 76.19, *SD_within_* = 11.93, *SD_between_* = 18.50). They also reported high levels of daily life satisfaction (*M* = 62.91, *SD_within_* = 13.72, *SD_between_* = 19.98). As shown in Table S11, the moderated multilevel regression analyses showed no evidence for an association between forecasting accuracy and either coping or life-satisfaction. Experienced affect significantly predicted coping and life-satisfaction in the expected directions: positive affect predicted greater coping and life-satisfaction, while negative affect predicted worse coping and lower life-satisfaction. But neither forecasted affect, nor the interaction between forecasted and experienced affect, predicted coping or life-satisfaction. These results held for both (1) momentary and retrospective ratings of experienced affect, and (2) momentary and retrospective emotion-focused coping, as well as when controlling for yesterday’s experienced affect (Table S12).

We replicated these models using multilevel RSA, which similarly showed no support for a congruence effect (see Figure S2). These findings were consistent across positive and negative affect, experienced affect operationalization (momentary ratings, retrospective ratings), and outcome (coping, life satisfaction). Full results including response surface and multilevel polynomial coefficients appear in Tables S13-S16. The multilevel polynomial coefficients consistently showed experienced affect was the only significant predictor of emotional benefits. In sum, our hypothesis that forecasting accuracy would predict greater emotion-focused coping and daily life satisfaction was not supported.

These findings suggest that perhaps being accurate is not functional, per se, but having biased forecasts is. One possibility is that by forecasting a stronger emotional signal, people might be better able to determine whether and how they should use future-oriented regulation strategies, like situation selection or modification (Floerke et al., 2017). In the case of negative affect, however, expecting days or situations to be worse than they are could lead to an over-reliance on avoidance as an emotion regulation strategy. Thus, perhaps there are bounds around when and how affective forecasts should inform regulation strategy selection. Future work should examine whether and how affective forecasts shape emotion regulation strategy selection, particularly in everyday life.

## Table S1

### Multilevel Regression Model Estimates: Association Between Forecasting Accuracy and Emotion-Focused Coping and Daily Life Satisfaction

|  | **Positive affect** | | | **Negative affect** | | |
| --- | --- | --- | --- | --- | --- | --- |
| *Outcome and predictors* | *Estimate (SE)* | *95% CI* | *p* | *Estimate (SE)* | *95% CI* | *p* |
| Momentary emotion-focused coping | |  |  |  |  |  |
| Intercept | -0.03 (0.05) | -0.13 – 0.07 | .573 | -0.03 (0.05) | -0.14 – 0.07 | .528 |
| Daily forecasted affect | 0.01 (0.01) | -0.02 – 0.03 | .630 | -0.01 (0.01) | -0.03 – 0.01 | .543 |
| Experienced momentary affect | **0.23 (0.01)** | **0.21 – 0.26** | **<.001** | **-0.23 (0.01)** | **-0.25 – -0.21** | **<.001** |
| Forecasting accuracy (interaction) | -0.00 (0.01) | -0.02 – 0.02 | .665 | -0.00 (0.01) | -0.02 – 0.02 | .968 |
| Daily emotion-focused coping | |  |  |  |  |  |
| Intercept | -0.04 (0.06) | -0.16 – 0.07 | .453 | -0.04 (0.06) | -0.16 – 0.07 | .463 |
| Daily forecasted affect | 0.00 (0.02) | -0.03 – 0.04 | .812 | -0.00 (0.02) | -0.04 – 0.03 | .904 |
| Experienced daily affect | **0.19 (0.02)** | **0.14 – 0.24** | **<.001** | **-0.22 (0.02)** | **-0.26 – -0.17** | **<.001** |
| Forecasting accuracy (interaction) | 0.03 (0.02) | -0.00 – 0.06 | .095 | -0.01 (0.01) | -0.04 – 0.02 | .388 |
| Daily life satisfaction |  |  |  |  |  |  |
| Intercept | -0.03 (0.06) | -0.14 – 0.09 | .643 | -0.04 (0.06) | -0.15 – 0.08 | .546 |
| Daily forecasted affect | 0.03 (0.02) | -0.00 – 0.06 | .087 | -0.01 (0.02) | -0.05 – 0.02 | .492 |
| Experienced daily affect | **0.28 (0.02)** | **0.24 – 0.32** | **<.001** | **-0.25 (0.02)** | **-0.30 – -0.21** | **<.001** |
| Forecasting accuracy (interaction) | 0.00 (0.02) | -0.03 – 0.03 | .986 | 0.01 (0.02) | -0.03 – 0.04 | .703 |

Note: in these models, forecasting accuracy was represented by the interaction between daily forecasted and experienced daily (retrospective estimate), or momentary, affect.

## Table S2

### Results of the Regression Models Testing Forecasting Accuracy Consequences Including Yesterday’s Affect as a Control Variable

|  | **Positive affect** | | | **Negative affect** | | |
| --- | --- | --- | --- | --- | --- | --- |
| *Outcome and predictors* | *Estimate (SE)* | *95% CI* | *p* | *Estimate (SE)* | *95% CI* | *p* |
| Momentary emotion focused coping | | |  |  |  |  |
| Intercept | -0.03 (0.05) | -0.14 – 0.07 | .533 | -0.04 (0.05) | -0.14 – 0.07 | .495 |
| Daily forecasted affect | 0.01 (0.01) | -0.02 – 0.03 | .633 | -0.01 (0.01) | -0.03 – 0.01 | .321 |
| Experienced momentary affect | **0.23 (0.01)** | **0.20 – 0.26** | **<.001** | -0.23 (0.01) | -0.25 – -0.21 | **<.001** |
| Yesterday’s experienced affect (control) | 0.00 (0.01) | -0.02 – 0.02 | .942 | 0.01 (0.01) | -0.01 – 0.03 | .286 |
| Forecasting accuracy (forecasted x experienced affect) | -0.00 (0.01) | -0.02 – 0.01 | .580 | -0.00 (0.01) | -0.02 – 0.02 | .937 |
| Daily emotion focused coping | | |  |  |  |  |
| Intercept | -0.04 (0.06) | -0.16 – 0.07 | .453 | -0.04 (0.06) | -0.16 – 0.07 | .463 |
| Daily forecasted affect | 0.00 (0.02) | -0.04 – 0.04 | .942 | 0.00 (0.02) | -0.03 – 0.04 | .870 |
| Experienced daily affect | **0.19 (0.03)** | **0.14 – 0.24** | **<.001** | **-0.22 (0.02)** | **-0.26 – -0.17** | **<.001** |
| Yesterday’s experienced affect (control) | 0.01 (0.02) | -0.02 – 0.05 | .517 | -0.01 (0.02) | -0.05 – 0.02 | .489 |
| Forecasting accuracy (predicted x experienced affect) | 0.03 (0.02) | -0.00 – 0.06 | .095 | -0.01 (0.01) | -0.04 – 0.02 | .371 |
| Daily life satisfaction |  |  |  |  |  |  |
| Intercept | -0.03 (0.06) | -0.15 – 0.09 | .632 | -0.04 (0.06) | -0.16 – 0.07 | .472 |
| Daily forecasted affect | 0.03 (0.02) | -0.01 – 0.06 | .121 | -0.02 (0.02) | -0.06 – 0.02 | .301 |
| Experienced daily affect | **0.28 (0.02)** | **0.24 – 0.32** | **<.001** | **-0.25 (0.02)** | **-0.30 – -0.21** | **<.001** |
| Yesterday’s experienced affect (control) | 0.00 (0.02) | -0.03 – 0.04 | .830 | 0.01 (0.02) | -0.02 – 0.05 | .413 |
| Forecasting accuracy (predicted x experienced affect) | 0.00 (0.02) | -0.03 – 0.03 | .960 | 0.01 (0.01) | -0.01 – 0.04 | .319 |

| Figure S2Response Surfaces for Multilevel RSA Testing Whether Congruence Between Forecasted and Experienced Positive Affect (PA) or Negative Affect (NA) Predicted Emotion-Focused Coping (Panels A, C, E, G) or Daily Life Satisfaction (Panels B, D, F, H) | | | |
| --- | --- | --- | --- |
| 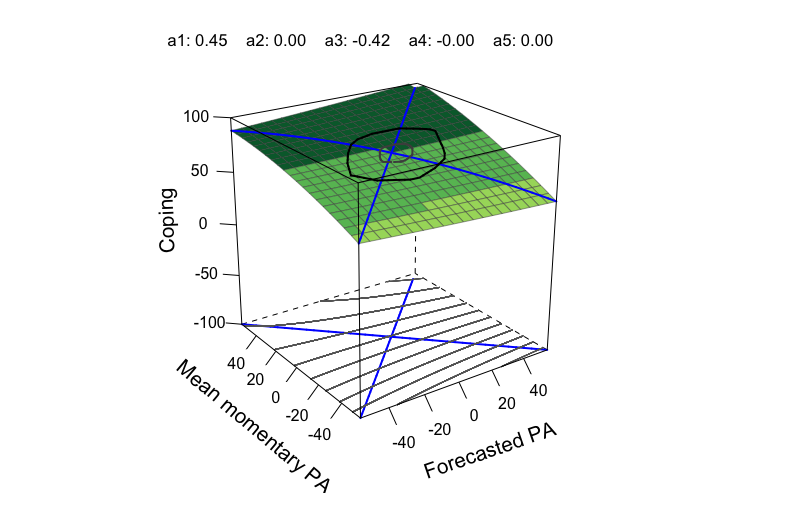A | 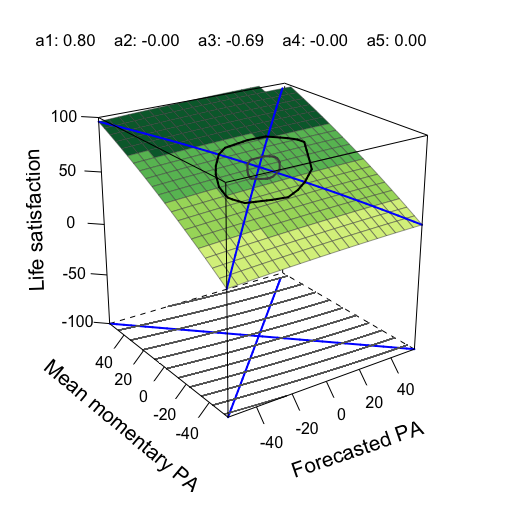B | 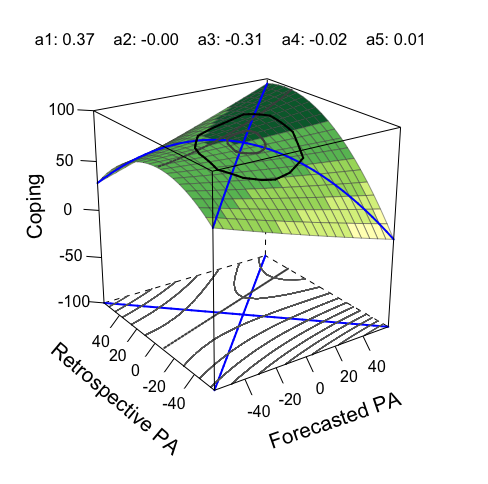C | D  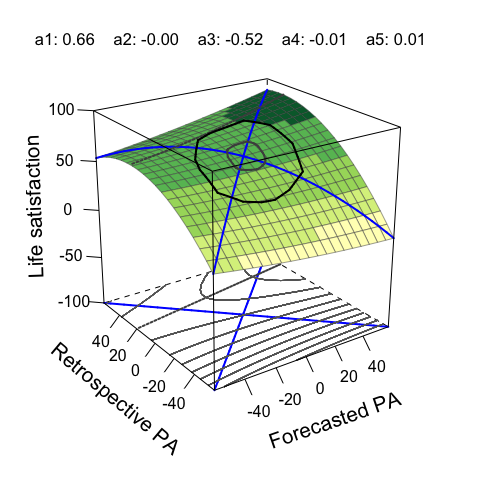 |
| 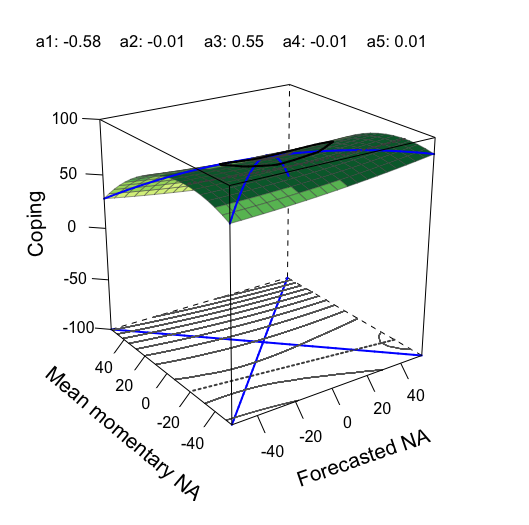E | 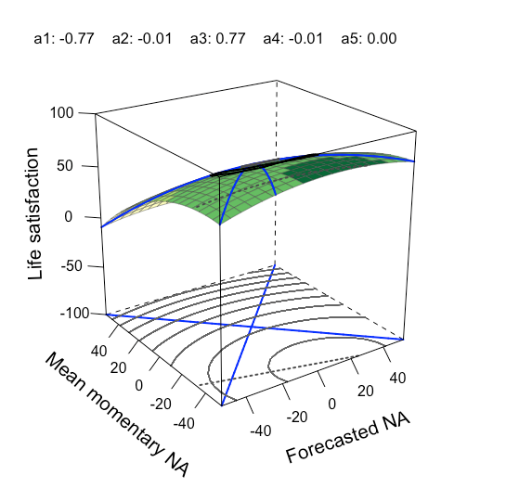F | 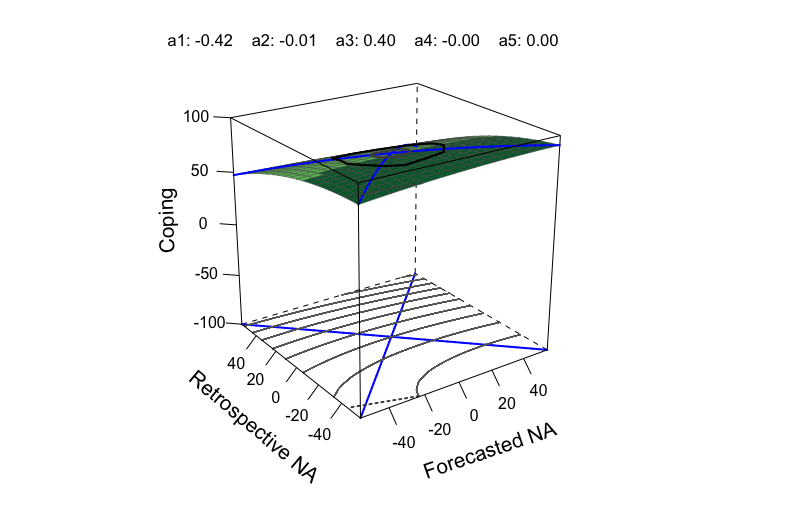G | H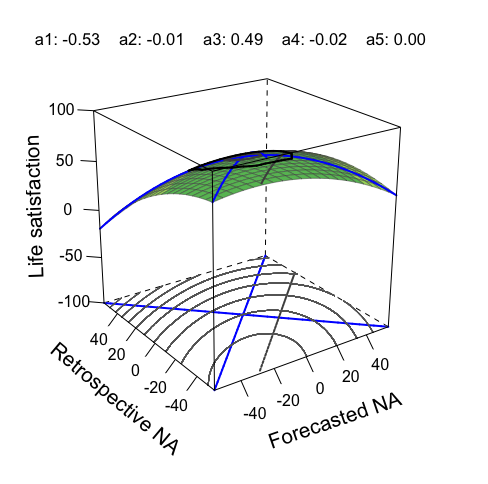 |

# Full Results of the Response Surface Analyses Testing Whether Forecasting Accuracy Confers Emotional Benefits

Tables S13-S16 display the full results for the response surface analyses. These analyses tested congruence hypotheses: that matches between forecasted and experienced affect predicted the highest levels of emotion-focused coping or daily life satisfaction. We found no support for congruence hypotheses in any of the models. Accompanying figures appear in the main paper.

## Table S3

### Response surface and multilevel polynomial coefficients for the models testing whether congruence between forecasted and experienced (day mean) positive affect predicts emotion-focused coping or daily life satisfaction.

|  | Emotion-focused coping  (Figure S2A) | | | Daily life satisfaction  (Figure S2B) | | |
| --- | --- | --- | --- | --- | --- | --- |
| Parameter | *b* | *SE* | *p* | *b* | *SE* | *p* |
| Response surface coefficients | |  |  |  |  |  |
| *â_1_* | 0.452 | 0.058 | <.001 | 0.796 | 0.064 | <.001 |
| *â_2_* | 0.00 | 0.004 | .980 | -0.002 | 0.004 | .667 |
| *â_3_* | -0.419 | 0.077 | <.001 | -0.687 | 0.084 | <.001 |
| *â_4_* | -0.004 | 0.006 | .523 | -0.002 | 0.006 | .782 |
| *â_5_* | 0.002 | 0.004 | .570 | 0.001 | 0.004 | .794 |
| Multilevel polynomial coefficients | |  |  |  |  |  |
| Intercept | 76.57 | 1.38 | **<.001** | 63.48 | 1.51 | **<.001** |
| *X* (Forecasted positive affect) | 0.02 | 0.03 | .642 | 0.05 | 0.04 | .156 |
| *Y* (Experienced positive affect – day mean) | 0.44 | 0.06 | **<.001** | 0.74 | 0.06 | **<.001** |
| *X^2^* | 0.00 | 0.00 | .920 | -0.00 | 0.00 | .847 |
| *XY* | 0.00 | 0.00 | .571 | -0.00 | 0.00 | .987 |
| *Y^2^* | -0.00 | 0.00 | .549 | -0.00 | 0.00 | .687 |

Note: In these models, experienced affect was operationalised by the day-mean of momentary positive affect ratings. A congruence hypothesis is met when *â_1,_ â_2_*_,_ *â_3,_* and *â_5_* are not significant, and *â_4_* is significant.

## Table S4

### Response surface and multilevel polynomial coefficients for the models testing whether congruence between forecasted and experienced (retrospective) positive affect predicts emotion-focused coping or daily life satisfaction.

|  | Emotion-focused coping  (Figure S2C) | | | Daily life satisfaction  (Figure S2D) | | |
| --- | --- | --- | --- | --- | --- | --- |
| Parameter | *b* | *SE* | *p* | *b* | *SE* | *p* |
| Response surface coefficients | |  |  |  |  |  |
| *â_1_* | 0.369 | 0.052 | <.001 | 0.661 | 0.048 | <.001 |
| *â_2_* | -0.001 | 0.003 | .695 | -0.004 | 0.003 | .165 |
| *â_3_* | -0.312 | 0.067 | <.001 | -0.516 | 0.069 | <.001 |
| *â_4_* | -0.020 | 0.005 | <.001 | -0.011 | 0.005 | .016 |
| *â_5_* | 0.011 | 0.003 | <.001 | 0.010 | 0.003 | .001 |
| Multilevel polynomial coefficients | |  |  |  |  |  |
| Intercept | 77.33 | 1.35 | **<.001** | 64.04 | 1.48 | **<.001** |
| *X* (Forecasted positive affect) | 0.03 | 0.03 | .399 | 0.07 | 0.04 | .047 |
| *Y* (Experienced positive affect – retrospective) | 0.34 | 0.05 | **<.001** | 0.59 | 0.05 | **<.001** |
| *X^2^* | -0.00 | 0.00 | .965 | 0.00 | 0.00 | .665 |
| *XY* | 0.01 | 0.00 | **.001** | 0.00 | 0.00 | .183 |
| *Y^2^* | -0.01 | 0.00 | **<.001** | -0.01 | 0.00 | **<.001** |

Note: In these models, experienced affect was operationalised by retrospective positive affect ratings. A congruence hypothesis is met when *â_1,_ â_2_*_,_ *â_3,_* and *â_5_* are not significant, and *â_4_* is significant.

## Table S5

### Response surface and multilevel polynomial coefficients for the models testing whether congruence between forecasted and experienced (day mean) negative affect predicts emotion-focused coping or daily life satisfaction.

|  | Emotion-focused coping  (Figure S2E) | | | Daily life satisfaction  (Figure S2F) | | |
| --- | --- | --- | --- | --- | --- | --- |
| Parameter | *b* | *SE* | *p* | *b* | *SE* | *p* |
| Response surface coefficients | |  |  |  |  |  |
| *â_1_* | -0.583 | 0.074 | <.001 | -0.768 | 0.093 | <.001 |
| *â_2_* | -0.013 | 0.004 | .004 | -0.015 | 0.005 | .004 |
| *â_3_* | 0.547 | 0.095 | <.001 | 0.775 | 0.112 | <.001 |
| *â_4_* | -0.006 | 0.006 | .307 | -0.011 | 0.008 | .165 |
| *â_5_* | 0.011 | 0.004 | .009 | 0.004 | 0.005 | .490 |
| Multilevel polynomial coefficients | |  |  |  |  |  |
| Intercept | 75.02 | 1.43 | **<.001** | 61.37 | 1.55 | **<.001** |
| *X* (Forecasted affect) | -0.02 | 0.05 | .704 | 0.003 | 0.06 | .953 |
| *Y* (Experienced affect – retrospective) | -0.56 | 0.07 | **<.001** | -0.77 | 0.09 | **<.001** |
| *X^2^* | 0.00 | 0.00 | .670 | -0.00 | 0.00 | .065 |
| *XY* | -0.00 | 0.00 | .393 | -0.00 | 0.00 | .638 |
| *Y^2^* | -0.01 | 0.00 | **.004** | -0.01 | 0.00 | **.066** |

Note: In these models, experienced affect was operationalised by the day mean of momentary negative affect ratings. A congruence hypothesis is met when *â_1,_ â_2_*_,_ *â_3,_* and *â_5_* are not significant, and *â_4_* is significant.

## Table S6

### Response surface and multilevel polynomial coefficients for the models testing whether congruence between forecasted and experienced (retrospective) negative affect predicts emotion-focused coping or daily life satisfaction.

|  | Emotion-focused coping  (Figure S2G) | | | Daily life satisfaction  (Figure S2H) | | |
| --- | --- | --- | --- | --- | --- | --- |
| Parameter | *b* | *SE* | *p* | *b* | *SE* | *p* |
| Response surface coefficients | |  |  |  |  |  |
| *â_1_* | -0.418 | 0.051 | <.001 | -0.532 | 0.060 | <.001 |
| *â_2_* | -0.005 | 0.003 | .085 | -0.006 | 0.003 | .067 |
| *â_3_* | 0.399 | 0.067 | <.001 | 0.491 | 0.074 | <.001 |
| *â_4_* | -0.002 | 0.004 | .604 | -0.018 | 0.005 | <.001 |
| *â_5_* | 0.002 | 0.003 | .405 | 0.000 | 0.003 | .939 |
| Multilevel polynomial coefficients | |  |  |  |  |  |
| Intercept | 76.30 | 1.38 | **<.001** | 63.69 | 1.48 | **<.001** |
| *X* (Forecasted affect) | -0.01 | 0.04 | .804 | -0.02 | 0.05 | .660 |
| *Y* (Experienced affect – retrospective) | -0.41 | 0.05 | **<.001** | -0.51 | 0.05 | **<.001** |
| *X^2^* | -0.00 | 0.00 | .705 | -0.01 | 0.00 | **.009** |
| *XY* | -0.00 | 0.00 | .540 | 0.01 | 0.00 | **.043** |
| *Y^2^* | -0.00 | 0.00 | .116 | -0.01 | 0.00 | **.003** |

Note: In these models, experienced affect was operationalised by retrospective daily negative affect ratings. A congruence hypothesis is met when *â_1,_ â_2_*_,_ *â_3,_* and *â_5_* are not significant, and *â_4_* is significant.

# Supplementary Forecasting Accuracy Analyses – Study 1

## Table S7

|  | **Momentary Experienced Affect** | | | **Weekly Retrospective Experienced Affect** | | |
| --- | --- | --- | --- | --- | --- | --- |
|  | *Estimate (SE)* | *95% CI* | *p* | *Estimate (SE)* | *95% CI* | *p* |
| Positive affect | |  |  |  |  |  |
| Intercept | 42.40 (0.81) | 40.79 – 44.00 | <.001 | 49.13 (1.60) | 45.96 – 52.29 | **<.001** |
| Weekly Forecasted Affect | **0.44 (0.04)** | **0.37 – 0.52** | **<.001** | 0.12 (0.08) | -0.03 – 0.28 | .125 |
| Negative affect | |  |  |  |  |  |
| Intercept | 23.18 (0.81) | 21.58 – 24.79 | <.001 | 32.49 (1.85) | 28.84 – 36.14 | **<.001** |
| Weekly Forecasted Affect | **0.51 (0.04)** | **0.44 – 0.59** | **<.001** | -0.00 (0.08) | -0.17 – 0.16 | .984 |

### Model Estimates: Relative Accuracy of Weekly Affective Forecasts – 2 item scales

*Note.* The models with momentary experienced affect as the outcome were multilevel models. The models with weekly retrospective affect as the outcome were not multilevel models. Bold indicates significant fixed-effects for predictors of interest.

## Table S8

### Results of the Daily Forecasting Accuracy Models Including Yesterday’s Affect as a Control Variable

|  | **Momentary Experienced Affect** | | | **Retrospective Experienced Affect** | | |
| --- | --- | --- | --- | --- | --- | --- |
|  | *Estimate (SE)* | *95% CI* | *p* | *Estimate (SE)* | *95% CI* | *p* |
| Positive affect |  |  |  |  |  |  |
| Intercept | 43.38 (0.41) | 42.56 – 44.20 | **<.001** | 44.45 (0.45) | 43.57 – 45.34 | **<.001** |
| Daily forecasted affect (person-mean centered) | **0.26 (0.02)** | **0.21 – 0.30** | **<.001** | **0.31 (0.03)** | **0.25 – 0.37** | **<.001** |
| Yesterday’s experienced affect (lagged retrospective) | 0.03 (0.02) | -0.01 – 0.07 | .174 | -0.01 (0.03) | -0.07 – 0.05 | 0.762 |
| Person mean daily forecasted affect (grand-mean centered) | **0.84 (0.03)** | **0.78 – 0.89** | **<.001** | **0.92 (0.03)** | **0.87 – 0.98** | **<.001** |
| Negative affect |  |  |  |  |  |  |
| Intercept | 23.73 (0.45) | 22.84 – 24.61 | **<.001** | 29.01 (0.48) | 28.07 – 29.96 | **<.001** |
| Daily forecasted affect (person-mean centered) | **0.23 (0.03)** | **0.18 – 0.29** | **<.001** | **0.28 (0.04)** | **0.20 – 0.37** | **<.001** |
| Yesterday’s experienced affect (lagged retrospective) | 0.03 (0.02) | -0.01 – 0.08 | 0.177 | -0.02 (0.03) | -0.08 – 0.05 | -0.02 (0.03) |
| Person mean daily forecasted affect (grand-mean centered) | **0.79 (0.03)** | **0.74 – 0.84** | **<.001** | **0.92 (0.03)** | **0.86 – 0.97** | **<.001** |

# Between and within-person correlations for key Study 1 variables

Table S9 displays correlations between the forecasted and experienced affect ratings made at the start and the end of the week, and the other key variables. These are between-person correlations because weekly affect ratings were made once and are a person-level variable. Table S10 displays between- and within-person correlations for the remaining variables.

## Table S9

### Between-person Correlations (r, [95% CI]) Between Weekly Forecasted, Weekly Experienced Affect, and All Other Variables

|  | Weekly forecasted positive affect | Weekly forecasted negative affect | Weekly experienced positive affect | Weekly experienced negative affect |
| --- | --- | --- | --- | --- |
| Weekly forecasted positive affect | - |  |  |  |
| Weekly forecasted negative affect | -.43***  [-.54, -.32] | - |  |  |
| Weekly experienced positive affect | 0.12  [-.03, .27] | -0.04  [-.19, .12] | - |  |
| Weekly experienced negative affect | -0.14  [-.29, .02] | -0.01  [-.17, .15] | -.40***  [-.52, -.26] | - |
| Daily forecasted positive affect (lagged) | .64***  [.55, .71] | -.41***  [-.52, -.29] | 0.03  [-.13, .18] | -0.07  [-.22, .09] |
| Daily forecasted negative affect (lagged) | -.38***  [-.49, -.26] | .67***  [.59, .74] | -0.08  [-.23, .08] | 0.08  [-.08, .24] |
| Daily experienced positive affect | .64***  [.55, .71] | -.38***  [-.49, -.26] | -0.02  [-.18, .14] | 0.02  [-.14, .17] |
| Daily experienced negative affect | -.36***  [-.47, -.24] | .63***  [.55, .71] | -0.03  [-.18, .13] | 0.03  [-.13, .18] |
| Momentary positive affect | .61***  [.52, .69] | -.33***  [-.45, -.21] | 0.03  [-.13, .28] | 0.03  [-.13, .18] |
| Momentary negative affect | -.31***  [-.42, -.18] | .60***  [.50, .68] | -0.06  [-.22, .10] | 0.04  [-.12, .19] |

Note: *** indicates *p* <.001

| Table S10Within and Between Person Correlations (r, [95%CI]) For Day- and Momentary-Level Variables | | | | | | |  |
| --- | --- | --- | --- | --- | --- | --- | --- |
|  | Daily forecasted positive affect (lagged) | Daily forecasted negative affect (lagged) | Daily experienced positive affect | Daily experienced negative affect | Momentary positive affect | Momentary negative affect | |
| Daily forecasted positive affect (lagged) |  | -.44  [-.49, -.40] | .32  [.27, .37] | -.17  [-.23, -.12] | .16  [.14, .19] | -.12  [-.09, -.14] | |
| Daily forecasted negative affect (lagged) | -.45  [-.55, -.34] |  | -.23  [-.28, -.17] | .28  [.22, .33] | -.09  [-.07, -.12] | .21  [.19, .23] | |
| Daily experienced positive affect | .93  [.91, .95] | -.41  [-.52, -.29] |  | -.48  [-.52, -.44] | .40  [.38, .42] | -.29  [-.27, -.31] | |
| Daily experienced negative affect | -.40  [-.51, -.28] | .92  [.90, .94] | -.42  [-.53, -.30] |  | -.28  [-.30, -.26] | .40  [.38, .42] | |
| Momentary positive affect | .91  [.88, .93] | -.34  [-.46, -.21] | .93  [.90, .94] | -.32  [-.44, -.20] |  | -.55  [-.54, -.56] | |
| Momentary negative affect | -.35  [-.46, -.22] | .91  [.88, .93] | -.34  [-.46, -.22] | .94  [.93, .96] | -.30  [-.42, -.17] |  | |

*Note*. All correlations significant at *p* < .001. Between-person correlations are below the diagonal line. Within-person correlations are above the diagonal line. Correlations calculated using the psych package in *R* (Revelle, 2021).

# Additional Supplementary Forecasting Accuracy Analyses – Study 1

## Table S11

| **Positive** | **Enthusiastic** | | | **Excited** | | | **Relaxed** | | | **Peaceful** | | |
| --- | --- | --- | --- | --- | --- | --- | --- | --- | --- | --- | --- | --- |
| *Predictors* | *Estimate (SE)* | *95% CI* | *p* | *Estimate (SE)* | *95% CI* | *p* | *Estimate (SE)* | *95% CI* | *p* | *Estimate (SE)* | *95% CI* | *p* |
| Intercept | 40.94 (0.67) | 39.62 – 42.27 | **<.001** | 39.22 (0.66) | 37.92 – 40.52 | **<.001** | 49.43 (0.56) | 48.32 – 50.54 | **<.001** | 48.29 (0.56) | 47.19 –  49.38 | **<.001** |
| Forecasted affect | **0.19 (0.03)** | **0.13 – 0.24** | **<.001** | **0.25 (0.03)** | **0.18 – 0.32** | **<.001** | **0.25 (0.03)** | **0.18 –**  **0.32** | **<.001** | **0.20 (0.03)** | **0.14 – 0.27** | **<.001** |
| Person mean forecasted affect | **0.97 (0.04)** | **0.90 – 1.04** | **<.001** | **0.92 (0.03)** | **0.85 – 0.99** | **<.001** | **0.91 (0.03)** | **0.85 –**  **0.98** | **<.001** | **0.91 (0.03)** | **0.85 – 0.97** | **<.001** |
| **Negative** | **Anxious** | | | **Irritated** | | | **Sad** | | | **Dull** | | |
| *Predictors* | *Estimate (SE)* | *95% CI* | *p* | *Estimate (SE)* | *95% CI* | *p* | *Estimate (SE)* | *95% CI* | *p* | *Estimate (SE)* | *95% CI* | *p* |
| Intercept | 35.87 (0.65) | 34.59 – 37.15 | **<.001** | 29.89 (0.70) | 28.51 – 31.28 | **<.001** | 23.02 (0.61) | 21.82 – 24.22 | **<.001** | 27.69 (0.68) | 26.36 –  29.03 | **<.001** |
| Forecasted Affect | **0.26 (0.04)** | **0.19 – 0.33** | **<.001** | **0.31 (0.04)** | **0.23 – 0.38** | **<.001** | **0.18 (0.04)** | **0.10 –**  **0.26** | **<.001** | **0.15 (0.04)** | **0.07 – 0.22** | **<.001** |
| Person mean forecasted affect | **0.87 (0.03)** | **0.81 – 0.93** | **<.001** | **0.87 (0.04)** | **0.79 – 0.94** | **<.001** | **0.97 (0.03)** | **0.91 –**  **1.03** | **<.001** | **0.96 (0.03)** | **0.89 – 1.02** | **<.001** |

### Models Estimating Relative Accuracy in Forecasting Specific Emotions – Study 1: Retrospective Experienced Affect Ratings

*Note.* Forecasted affect represents the within person effect and was person mean centered. Person mean forecasted affect represents the between person effect and was grand mean centered.

## Table S12

### Models Estimating Absolute Accuracy in Forecasting Specific Emotions – Study 1: Day Mean Momentary Affect Ratings

| **Positive** | **Enthusiastic** | | | **Excited** | | | **Relaxed*** | | | **Peaceful*** | | |
| --- | --- | --- | --- | --- | --- | --- | --- | --- | --- | --- | --- | --- |
|  | *Estimate (SE)* | *95% CI* | *p* | *Estimate (SE)* | *95% CI* | *p* | *Estimate (SE)* | *95% CI* | *p* | *Estimate (SE)* | *95% CI* | *p* |
| Intercept | 38.36 (1.23) | 35.94 – 40.79 | **<.001** | 35.66 (1.23) | 33.23 – 38.08 | **<.001** | 50.26 (1.15) | 47.98 – 52.53 | **<.001** | 48.94 (1.24) | 46.50 – 51.39 | **<.001** |
| Forecasted Affect | **7.77 (0.57)** | **6.66 – 8.88** | **<.001** | **7.65 (0.58)** | **6.52 – 8.79** | **<.001** | **-1.89 (0.60)** | **-3.07 – -0.71** | **.002** | -0.27 (0.58) | -1.41 – 0.87 | .644 |
| **Negative** | **Anxious** | | | **Irritated** | | | **Sad** | | | **Dull** | |  |
|  | *Estimate (SE)* | *95% CI* | *p* | *Estimate (SE)* | *95% CI* | *p* | *Estimate (SE)* | *95% CI* | *p* | *Estimate (SE)* | *95% CI* | *p* |
| Intercept | 28.85 (1.41) | 26.08 – 31.62 | **<.001** | 22.61 (1.16) | 20.32 – 24.90 | **<.001** | 17.87 (1.29) | 15.33 – 20.40 | **<.001** | 26.16 (1.37) | 23.46 – 28.86 | **<.001** |
| Forecasted Affect | **9.13 (0.59)** | **7.97 – 10.29** | **<.001** | **6.58 (0.58)** | **5.44 – 7.72** | **<.001** | **4.52 (0.53)** | **3.47 – 5.56** | **<.001** | **2.63 (0.57)** | **1.51 – 3.75** | **<.001** |

*Note.* The intercept represents mean levels of experienced affect, and the estimate represents the difference between experienced and forecasted affect levels. Positive estimates represent overestimation and negative estimates represents underestimation. *indicates where findings differ relative to the main analyses for mean positive/negative affect.

## Table S13

### Models Estimating Absolute Accuracy in Forecasting Specific Emotions – Study 1: Retrospective Experienced Affect Ratings

| **Positive** | **Enthusiastic** | | | **Excited** | | | **Relaxed** | | | **Peaceful** | | |
| --- | --- | --- | --- | --- | --- | --- | --- | --- | --- | --- | --- | --- |
|  | *Estimate (SE)* | *95% CI* | *p* | *Estimate (SE)* | *95% CI* | *p* | *Estimate (SE)* | *95% CI* | *p* | *Estimate (SE)* | *95% CI* | *p* |
| Intercept | 41.28 (1.32) | 38.67 – 43.89 | **<.001** | 39.48 (1.31) | 36.90 – 42.06 | **<.001** | 49.34 (1.24) | 46.90 –  51.77 | **<.001** | 48.09 (1.30) | 45.52 –  50.66 | **<.001** |
| Forecasted Affect | **4.84 (0.64)** | **3.59 – 6.08** | **<.001** | **3.94 (0.66)** | **2.64 – 5.23** | **<.001** | -0.99 (0.67) | -2.31 – 0.32 | .138 | 0.57 (0.65) | -0.70 –  1.84 | .381 |
| **Negative** | **Anxious** | | | **Irritated** | | | **Sad** | | | **Dull** | | |
|  | *Estimate (SE)* | *95% CI* | *p* | *Estimate (SE)* | *95% CI* | *p* | *Estimate (SE)* | *95% CI* | *p* | *Estimate (SE)* | *95% CI* | *p* |
| Intercept | 35.88 (1.53) | 32.87 – 38.89 | **<.001** | 29.98 (1.28) | 27.47 – 32.50 | **<.001** | 23.03 (1.40) | 20.27 –  25.79 | **<.001** | 28.54 (1.43) | 25.73 –  31.36 | **<.001** |
| Forecasted Affect | **2.13 (0.68)** | **0.79 – 3.47** | **.002** | -0.84 (0.69) | -2.19 – 0.50 | .219 | -0.72 (0.63) | -1.95 – 0.52 | .254 | 0.17 (0.66) | -1.14 –  1.47 | .803 |

*Note.* The intercept represents mean levels of experienced affect, and the estimate represents the difference between experienced and forecasted affect levels. Positive estimates represent overestimation and negative estimates represents underestimation

# Event-related peak positive and negative intensity ratings – Study 2

In the morning survey, after describing their nominated unpleasant event participants rated how negative and positive they expected the situation to make them feel at its most negative/positive point (from 1= not at all to 9 = extremely). Mean forecasted positive intensity ratings were 3.32 (*SD_within_ =* 1.56, *SD_between_* = 1.44) and negative intensity ratings were 4.50 (*SD_within_ =* 1.69, *SD_between_* = 1.37).

In the evening survey, participants rated how positive and negative the event made them feel at its most negative/positive point. Mean experienced positive intensity ratings were 4.04 (*SD_within_ =* 2.06, *SD_between_* = 1.68) and negative intensity ratings were 3.89 (*SD_within_ =* 2.08, *SD_between_* = 1.34).

# Supplementary Forecasting Accuracy Analyses – Study 2

When we re-ran the relative and absolute accuracy models using these intensity ratings, we replicated the main positive and negative affect findings. For the relative accuracy results, see Table S13. For absolute accuracy, participants underestimated how positive they would feel (Est.=-0.66, SE= 0.11, *p*<.001) and overestimated how negative they would feel (Est.= 0.58, SE= 0.11, *p*<.001) at the peak of the event.

|  | Experienced positive intensity | | | Experienced negative intensity | | |
| --- | --- | --- | --- | --- | --- | --- |
|  | *Estimate (SE)* | *95% CI* | *p* | *Estimate (SE)* | *95% CI* | *p* |
| Intercept | 4.26 (0.19) | 3.87 – 4.64 | **<.001** | 3.67 (0.15) | 3.38 – 3.96 | **<.001** |
| Forecasted intensity | **0.29 (0.07)** | **0.15 – 0.43** | **<.001** | **0.44 (0.06)** | **0.31 – 0.56** | **<.001** |

## Table S14

### Multilevel Model Estimates: Relative Accuracy of Event-Related Forecasts of Peak Intensity

## Table S15

### Models Estimating Relative Accuracy in Forecasting Specific Emotions for Unpleasant Events – Study 2

|  | **Positive Emotions** | | | **Negative Emotions** | | | | | | | | |
| --- | --- | --- | --- | --- | --- | --- | --- | --- | --- | --- | --- | --- |
|  | **Happiness** | | | **Sadness** | | | **Boredom** | | | **Embarrassment/Shame** | | |
| *Predictors* | *Estimate (SE)* | *95% CI* | *p* | *Estimate (SE)* | *95% CI* | *p* | *Estimate (SE)* | *95% CI* | *p* | *Estimate (SE)* | *95% CI* | *p* |
| Intercept | 1.96 (0.06) | 1.85 – 2.08 | **<.001** | 1.70 (0.05) | 1.61 – 1.80 | **<.001** | 1.73 (0.04) | 1.66 – 1.81 | **<.001** | 1.41 (0.05) | 1.32 – 1.50 | **<.001** |
| Forecasted Affect | 0.48 (0.06) | 0.35 – 0.60 | **<.001** | 0.45 (0.05) | 0.34 – 0.55 | **<.001** | 0.46 (0.04) | 0.39 – 0.53 | **<.001** | 0.32 (0.04) | 0.24 – 0.39 | **<.001** |
| Person mean forecasted affect | 0.41 (0.10) | 0.21 – 0.62 | **<.001** | 0.36 (0.07) | 0.22 – 0.50 | **<.001** | 0.25 (0.06) | 0.13 – 0.37 | **<.001** | 0.25 (0.08) | 0.10 – 0.40 | **.002** |
|  | **Relaxation** | | | **Anxiety/Fear** | | | **Disgust** | | | **Anger** | | |
| *Predictors* | *Estimate (SE)* | *95% CI* | *p* | *Estimate (SE)* | *95% CI* | *p* | *Estimate (SE)* | *95% CI* | *p* | *Estimate (SE)* | *95% CI* | *p* |
| Intercept | 1.63 (0.05) | 1.53 – 1.73 | **<.001** | 2.25 (0.06) | 2.12 – 2.37 | **<.001** | 1.34 (0.04) | 1.27 – 1.41 | **<.001** | 1.57 (0.05) | 1.48 – 1.66 | **<.001** |
| Forecasted Affect | 0.60 (0.06) | 0.48 – 0.73 | **<.001** | 0.48 (0.04) | 0.41 – 0.55 | **<.001** | 0.39 (0.04) | 0.32 – 0.46 | **<.001** | 0.36 (0.04) | 0.29 – 0.43 | **<.001** |
| Person mean forecasted affect | 0.36 (0.10) | 0.16 – 0.56 | **.001** | 0.19 (0.08) | 0.03 – 0.35 | **.021** | 0.29 (0.07) | 0.14 – 0.44 | **<.001** | 0.23 (0.08) | 0.07 – 0.39 | **.006** |

## Table S16

### Models Estimating Absolute Accuracy in Forecasting Specific Emotions for Unpleasant Events – Study 2

|  | **Positive Emotions** | | | **Negative Emotions** | | | | | | | | |
| --- | --- | --- | --- | --- | --- | --- | --- | --- | --- | --- | --- | --- |
|  | **Happiness** | | | **Sadness** | | | **Boredom** | | | **Embarrassment/Shame** | | |
| *Predictors* | *Estimate (SE)* | *95% CI* | *p* | *Estimate (SE)* | *95% CI* | *p* | *Estimate (SE)* | *95% CI* | *p* | *Estimate (SE)* | *95% CI* | *p* |
| Intercept | 1.93 (0.08) | 1.77 – 2.09 | **<.001** | 1.73 (0.08) | 1.56 – 1.89 | **<.001** | 1.71 (0.09) | 1.53 – 1.89 | **<.001** | 1.45 (0.07) | 1.30 – 1.60 | **<.001** |
| Forecasted Affect | **-0.16 (0.05)** | **-0.27 – -0.06** | **.002** | 0.10 (0.05) | -0.01 – 0.20 | **.064** | **0.32 (0.05)** | **0.22 – 0.43** | **<0.001** | **0.11 (0.04)** | **0.03 – 0.19** | **.007** |
|  | **Relaxation** | | | **Anxiety/Fear** | | | **Disgust** | | | **Anger** | | |
| *Predictors* | *Estimate (SE)* | *95% CI* | *p* | *Estimate (SE)* | *95% CI* | *p* | *Estimate (SE)* | *95% CI* | *p* | *Estimate (SE)* | *95% CI* | *p* |
| Intercept | 1.58 (0.07) | 1.44 – 1.73 | **<.001** | 2.29 (0.10) | 2.09 – 2.49 | **<.001** | 1.42 (0.07) | 1.28 – 1.56 | **<.001** | 1.67 (0.07) | 1.52 – 1.81 | **<.001** |
| Forecasted Affect | **-0.13 (0.05)** | **0.22 – -0.04** | **.005** | **0.26 (0.06)** | **0.14 – 0.37** | **<.001** | 0.04 (0.04) | -0.04 – 0.11 | .334 | **0.15 (0.05)** | **0.05 – 0.25** | **.003** |

*Note.* The intercept represents mean levels of experienced affect, and the estimate represents the difference between experienced and forecasted affect levels. Positive estimates represent overestimation and negative estimates represents underestimation.

| Between and within-person correlations for key Study 2 variablesTable S17Within and Between Person Correlations (r, [95%CI]) For Study 2 Variables | | | | | | | | |  |
| --- | --- | --- | --- | --- | --- | --- | --- | --- | --- |
|  | Forecasted positive affect | Forecasted negative affect | Experienced positive affect | Experienced negative affect | Forecasted peak positive intensity | Forecasted peak negative intensity | Experienced peak positive intensity | Experienced peak negative intensity | |
| Forecasted positive affect |  | -.42**  [-.48, -.35] | .50**  [.43, .55] | -.22**  [-.30, -.14] | .65**  [.60, .69] | -.46**  [-.51, -.40] | .30**  [.22, .37] | -.20**  [-.28, -.13] | |
| Forecasted negative affect | .04  [-.20, .27] |  | -.26**  [-.34, -.19] | .42**  [.35, .48] | -.41**  [-.47, -.34] | .63**  [.58, .67] | -.23**  [-.30, -.15] | .31**  [.23, .38] | |
| Experienced positive affect | .82**  [.73, .89] | -.01  [-.24, .23] |  | -.43**  [-.49, -.36] | .30**  [.23, .37] | -.25**  [-.32, -.17] | .65**  [.60, .69] | -.47**  [-.53, -.41] | |
| Experienced negative affect | .02  [-.22,.26] | .82**  [.73, .89] | -.13  [-.36, .11] |  | -.19**  [-.27, -.12] | .25**  [.17, .32] | -.50**  [-.56, -.44] | .64**  [.59, .68] | |
| Forecasted peak positive intensity | .79**  [.68, .87] | -.08  [-.31, .16] | .68**  [.53, .79] | -.06  [-.29, .18] |  | -.45**  [-.51, -.39] | .24**  [.17, .32] | -.17**  [-.24, -.09] | |
| Forecasted peak negative intensity | -.25*  [-.46, -.02] | .66**  [.51, .78] | -.23  [-.44,.01] | .48**  [.28, .65] | -.27*  [-.47, -.03] |  | -.18**  [-.26, -.10] | .33**  [.26, .40] | |
| Experienced peak positive intensity | .67**  [.51, .78] | -.05  [-.29, .19] | .84**  [.75, .90] | -.16  [-.39, .08] | .74**  [.60, .83] | -.16  [-.38, .08] |  | -.62**  [-.66, -.57] | |
| Experienced peak negative intensity | -.22  [-.44, .02] | .54**  [.35, .69] | -.43**  [-.61, -.22] | .67**  [.52, .79] | -.13  [-.36, .11] | .65**  [.49, .77] | -.39**  [-.58, -.17] |  | |

*Note*. **p* <.05, ***p* < .001. Between-person correlations are below the diagonal line. Within-person correlations are above the diagonal line. Correlations calculated using the psych package in *R* (Revelle, 2021)

# References

Carver, C. S., & Scheier, M. F. (2014). Dispositional optimism. *Trends in Cognitive Sciences*, *18*(6), 293–299. https://doi.org/10.1016/j.tics.2014.02.003

Erbas, Y., Kalokerinos, E. K., Kuppens, P., van Halem, S., & Ceulemans, E. (2021). Momentary Emotion Differentiation: The Derivation and Validation of an index to Study Within-Person Fluctuations in Emotion Differentiation. *Assessment*, 1073191121990089. https://doi.org/10.1177/1073191121990089

Floerke, V. A., Sands, M., Isaacowitz, D., Thomas, A. K., & Urry, H. L. (2017). Cloudy with a chance of feelings: Affective forecasting as a resource for situation selection across the lifespan [Preprint]. PsyArXiv. https://doi.org/10.31234/osf.io/mwtrp

Heller, D., Watson, D., & Ilies, R. (2006). The Dynamic Process of Life Satisfaction. *Journal of Personality*, *74*(5), 1421–1450. https://doi.org/10.1111/j.1467-6494.2006.00415.x

Le, B. M., Côté, S., Stellar, J., & Impett, E. A. (2020). The Distinct Effects of Empathic Accuracy for a Romantic Partner’s Appeasement and Dominance Emotions. *Psychological Science*, *31*(6), 607–622. https://doi.org/10.1177/0956797620904975

Nestler, S., Humberg, S., & Schönbrodt, F. D. (2019). Response surface analysis with multilevel data: Illustration for the case of congruence hypotheses. *Psychological Methods*, *24*(3), 291–308. https://doi.org/10.1037/met0000199

Neupert, S. D., & Bellingtier, J. A. (2019). Daily Stressor Forecasts and Anticipatory Coping: Age Differences in Dynamic, Domain-Specific Processes. *The Journals of Gerontology: Series B*, *74*(1), 17–28. <https://doi.org/10.1093/geronb/gby043>

Neupert, S. D., Neubauer, A. B., Scott, S. B., Hyun, J., & Sliwinski, M. J. (2019). Back to the Future: Examining Age Differences in Processes Before Stressor Exposure. *The Journals of Gerontology: Series B*, *74*(1), 1–6. https://doi.org/10.1093/geronb/gby074

Wang, Y.-R., Black, K. J., & Martin, A. (2020). Antecedents and outcomes of daily anticipated stress and stress forecasting errors. *Stress and Health*, *1–16*. https://doi.org/10.1002/smi.3044
